# Scalable quantum photonic platform based on site-controlled quantum dots coupled to circular Bragg grating resonators

**DOI:** 10.1038/s41377-026-02343-0

**Published:** 2026-06-02

**Authors:** Kartik Gaur, Avijit Barua, Sarthak Tripathi, Léo J. Roche, Hannah Wilksen, Alexander Steinhoff, Sam Baraz, Neha Nitin, Chirag C. Palekar, Aris Koulas-Simos, Imad Limame, Priyabrata Mudi, Sven Rodt, Christopher Gies, Stephan Reitzenstein

**Affiliations:** 1 Institut für Physik und Astronomie, Technische Universität Berlin, Hardenbergstraße 36, Berlin, 10623 Germany; 2 Institute for Physics, Carl von Ossietzky Universität Oldenburg, Oldenburg, 26129 Germany

**Keywords:** Lasers, LEDs and light sources, Quantum dots, Photonic devices

## Abstract

The scalable integration of solid-state quantum emitters into photonic nanostructures remains a central challenge for quantum photonic technologies. Here, we demonstrate a robust and streamlined integration strategy that tackles the long-standing issue of deterministic fabrication on randomly positioned self-assembled quantum dots (QDs), leveraging a buried-stressor-based site-controlled InGaAs QD platform. We show that this deterministic growth approach enables precise spatial alignment with circular Bragg grating (CBG) resonators for enhanced emission, eliminating the need for complex and time-consuming deterministic lithography techniques. We fabricated a 6 × 6 SCQD-CBG array with 100% device yield, with 35 devices falling within the radial-offset range where the simulated photon-extraction efficiency (PEE) exceeds 20%, underscoring the spatial precision and scalability of our fabrication concept. A systematically selected subset of five devices with varying radial displacements reveals clear offset-dependent trends in PEE, degree of linear polarization, spectral linewidth, and photon indistinguishability, thereby establishing quantitative bounds on spatial alignment tolerances. In the best-aligned QD-CBG device, we achieve a PEE of (47.1 ± 3.8)% (corresponding to an end-to-end system efficiency of 3.4%), a linewidth of (1.41 ± 0.22) GHz, a radiative decay lifetime of (0.80 ± 0.02) ns, a single-photon purity of (99.58 ± 0.18)%, and a Hong-Ou-Mandel two-photon interference visibility of (81 ± 5)% under quasi-resonant excitation at saturation power. We confirm our conceptual understanding of the effect of emitter-position dependent charge-noise fluctuations in terms of a quantum-optical model for the (quantum-)emission properties. The established nanofabrication platform provides a reproducible, lithography-compatible route to scalable, high-performance single-photon sources (SPS), offering a powerful alternative to conventional lithography-based deterministic integration techniques.

## Abstract (web summary)

Scalable marker-free integration of site-controlled quantum dots into circular Bragg grating resonators enables a high-yield array of bright, pure, and highly indistinguishable single-photon sources.

## Graphical abstract

**Figure:**
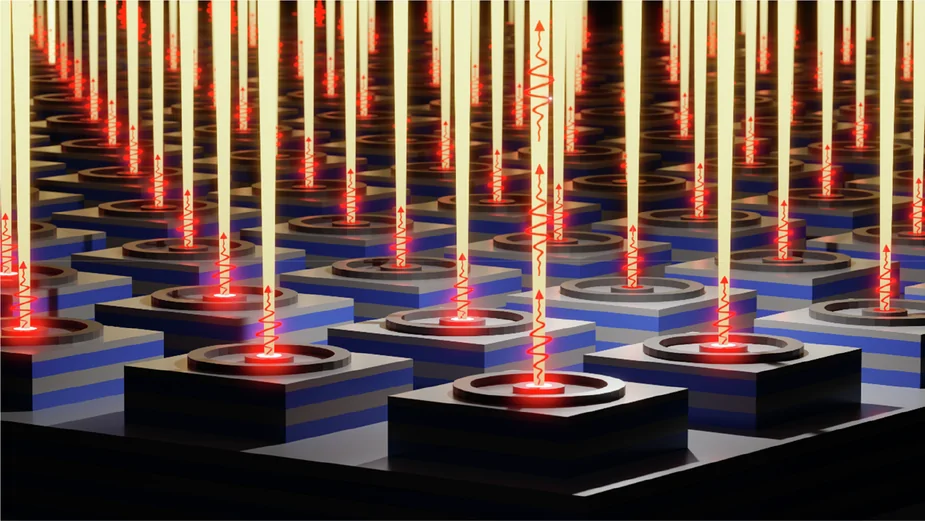

## Introduction

Semiconductor quantum dots (QDs) constitute one of the most powerful solid-state platforms for generating non-classical light, combining near-unity internal quantum efficiency, discrete energy levels, and compatibility with integrated photonic architectures^1–6^. Their potential for deterministic single-photon emission, photon indistinguishability, and electrical operability has positioned them at the forefront of photonic quantum information technology^4^, enabling proof-of-principle demonstrations of boson sampling^7,8^, quantum teleportation^9–11^, photonic quantum computing^6,12,13^, and elementary quantum networks^14–17^. Embedding QDs into optical microcavities, waveguides, or metastructures enables enhanced light-matter interaction, improving brightness, directionality, and coherence of the emitted photons^18–20^. However, achieving simultaneously high photon extraction efficiency (PEE) and near-unity indistinguishability remains nontrivial. Although a reduced radiative lifetime resulting from genuine Purcell enhancement can shorten the dephasing window and improve Hong-Ou-Mandel (HOM) visibility^21^, practical approaches to increasing brightness, such as stronger optical confinement or higher excitation powers, often introduce charge noise, spectral diffusion, and phonon-assisted processes that broaden the emission linewidth and reduce photon indistinguishability^22,23^. Careful optimization of the emitter environment and surrounding photonic structure, combined with resonant or quasi-resonant excitation schemes, can mitigate these effects and preserve coherence^24–26^. In this context, scalable arrays of QD-based single-photon sources (SPS) are highly desirable for large-scale quantum networks and photonic processors, where multiple indistinguishable emitters operating in parallel can enable on-chip entanglement distribution, multiplexed photon generation, and high-throughput quantum information processing^27,28^. However, the intrinsic randomness in the spatial and spectral positioning of self-assembled QDs fundamentally impedes large-scale integration^29,30^. Realizing spatial overlap between cavity modes and quantum emitters typically requires pre-characterization through cathodoluminescence (CL)^31,32^ or photoluminescence (PL) mapping^32–35^, followed by marker-based or in-situ lithography^36–38^, processing steps that introduce complexity, reduce throughput, and challenge scalability. In parallel, hybrid integration strategies based on transfer printing or “pick-and-place” assembly have also been explored, where pre-fabricated QDs or nanomembrane cavities are physically transferred onto target photonic circuits, but these approaches remain experimentally demanding and inherently limited in scalability^39,40^. Moreover, such approaches still rely on random QD positions, demand high-resolution imaging infrastructure and tight process control, restricting accessibility to well-equipped fabrication environments.

Site-controlled quantum dot (SCQD) platforms provide a pathway to overcome this barrier by enabling deterministic QD nucleation at lithographically defined locations^41,42^. Several techniques, such as surface patterning^43–47^ and selective area epitaxy^48,49^, have been explored to engineer QD placement with nanometer-scale precision. While these SCQD approaches have been used to demonstrate initial attempts at scalable integration into nanophotonic resonators^45,50,51^, their optical performance remained suboptimal, preventing them from reaching the benchmarks needed for scalable quantum-photonic systems. Beyond these, the buried-stressor approach has emerged as a promising candidate, leveraging strain fields from subsurface oxide apertures to guide QD formation at the center of patterned mesas^41,52,53^. This technique integrates seamlessly with standard planar growth protocols and, unlike surface-etched templates, avoids detrimental interface states that can compromise optical performance^54^. Furthermore, the stressor aperture size can be fine-tuned via oxidation control^55,56^, enabling systematic control over local QD density and site precision, which enables the fabrication of both quantum light sources^57^ and high-*β* microlasers^58^ based on SCQDs. While initial realizations of buried-stressor SCQDs faced challenges related to linewidth broadening and low radiative efficiency, recent progress in epitaxial optimization has led to substantial improvements in emitter quality, including reduced spectral diffusion and enhanced coherence, opening the door to their use in quantum photonic devices^57,59–61^.

Despite this, the integration of SCQDs into high-performance photonic structures has remained largely reliant on some form of emitter pre-localization or alignment^61^. In particular, achieving consistent spatial overlap between the quantum emitter and the cavity mode without reverting to feedback-based positioning has proven nontrivial. This challenge is especially pronounced in nanophotonic cavities such as circular Bragg gratings (CBGs), where even slight displacements of the emitter from the center of the cavity can markedly reduce PEE and can induce linear polarization of the emitted photons^62–64^. In particular, inverted pyramidal QDs grown in tetrahedral recesses on (111)B GaAs substrates have demonstrated excellent position control and deterministic coupling to membrane photonic crystal cavities^44,65^. However, this type of SCQDs is not directly compatible with the CBG cavity approach, and it will be challenging to adopt them for this SPS concept. Moreover, scalable integration strategies must remain compatible with standard and ideally industry-relevant nanofabrication workflows, minimize reliance on complex lithographic procedures, and ensure high optical quality across large device arrays. Bridging this gap requires an approach that combines deterministic emitter positioning with a fabrication process that is alignment-free, spectrally flexible, and scalable beyond a few isolated devices.

Here, we overcome this integration challenge by developing a marker-free, lithography-compatible nanofabrication platform that exploits the intrinsic spatial precision of buried-stressor-grown SCQDs for direct integration into CBG resonators. In this approach, deterministic QD nucleation at the center of the site-controlled growth (SCG) mesas enables device fabrication entirely through standard electron beam lithography (EBL), eliminating the need for emitter localization via CL or PL mapping or in-situ EBL typically required for randomly positioned QDs. CBGs are patterned directly on the predefined mesa grid without optical mapping, streamlining the process and enhancing device yield. The workflow operates entirely at room temperature and remains fully compatible with planar nanofabrication, circumventing any low-temperature alignment or processing steps. By decoupling cavity integration from emitter localization, it establishes a scalable, reproducible, and fabrication-efficient route toward wafer-level implementation of bright SPS with high photon indistinguishability.

## Results

In the following subsections, we introduce and discuss our scalable buried-stressor-based quantum device fabrication concept, presenting the epitaxial growth of SCQDs, the design and nanofabrication of SCQD-CBGs, the demonstration of scalable integration, and the systematic optical characterization of the resulting devices in combination with theoretical modeling of the quantum-emission properties.

### Site-controlled quantum dot growth via the buried-stressor approach

**Fig. 1:**
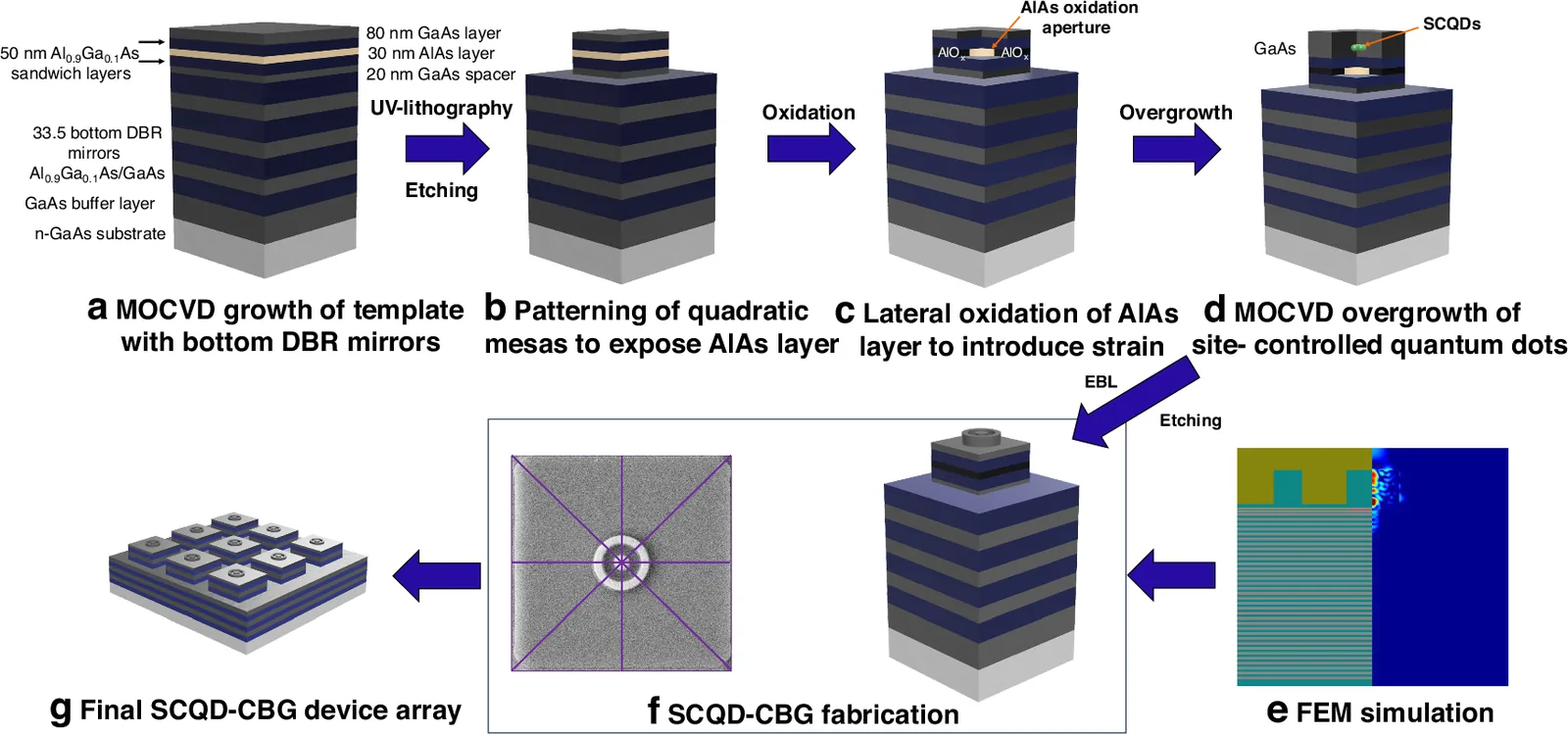
Schematic process flow for SCQD-CBG device fabrication. The fabrication pathway begins with MOCVD growth of a DBR template incorporating a buried AlAs stressor layer (**a**), followed by SCG-mesa definition through UV-lithography and etching (**b**) and lateral oxidation to form apertures at the center of the mesas (**c**). SCQDs are subsequently overgrown within a GaAs cavity by MOCVD (**d**). Prior to patterning, the CBG parameters were optimized through FEM simulations, as illustrated by the schematic device representation and the corresponding simulated electric field distribution (**e**). The CBGs with numerically optimized geometry are subsequently patterned on the SCG-mesas using EBL and etching (**f**), also shown in the representative SEM image of a completed SCQD-CBG device. This process yields an array of SCQD-CBG structures (**g**), ready for optical characterization

The growth of InGaAs SCQDs via the buried-stressor approach begins with metal-organic chemical vapor deposition (MOCVD) growth of a GaAs buffer layer, followed by 33.5 Al_0.9_Ga_0.1_As/GaAs DBR mirror pairs and a GaAs barrier layer. The buried-stressor layer comprises an AlAs core encapsulated between two Al_0.9_Ga_0.1_As layers, capped by a GaAs layer. The epitaxial template is lithographically patterned into square SCG-mesas, exposing the AlAs layer laterally. Selective oxidation of the AlAs layer forms an oxide-confined aperture that generates a localized tensile strain field, whose lateral extent and magnitude are governed by the oxidation time. Subsequent epitaxial overgrowth yields ultralow-density InGaAs QDs that nucleate deterministically at the center of the tensile strain maximum^53,55^, enabling spatially controlled emitter positioning without surface patterning. A thin GaAs capping layer and a final GaAs overgrowth complete the epitaxial structure. The complete fabrication workflow for realizing SCQD-CBG devices is illustrated in Fig. 1, outlining the sequential steps from DBR template growth and SCG-mesa patterning to SCQD overgrowth and final CBG patterning (discussed in subsection “Marker-free scalable integration of SCQDs into CBGs”), ultimately yielding a fully integrated device array.

### Optical assessment of SCG-mesas

The optical and spatial uniformity of the SCG-mesas was systematically evaluated prior to photonic cavity integration. Micro-photoluminescence (*μ*PL) spectroscopy was performed on over 200 individual SCG-mesas across a 5 × 5 mm^2^ sample piece to assess emission wavelength homogeneity and verify emitter yield. A detailed description of the experimental setup is provided in the Supplementary Information (SI) section 1 (Fig. S1). The measurements reveal consistent spectral characteristics across the device array, with the dominant excitonic transitions distributed between 925 nm and 945 nm. This wavelength spread reflects stable strain and composition control during the buried-stressor growth process. The absence of any pronounced spectral outliers underscores the uniformity of the growth environment across the mesoscale array and confirms the reproducibility of the site-selective nucleation process. Exemplary emission spectra of SCG-mesas, together with a statistical histogram illustrating the emission wavelength distribution of over 200 individual structures across the sample, are provided in Fig. S2 of the SI.

### Design and simulation of CBG resonators

The design parameters for the CBGs were numerically optimized by calculating electric field distributions and maximizing the PEE into a collection numerical aperture (NA) of 0.81, matching the objective lens used in our optical experiments. We performed simulations using the finite-element method (FEM) scattering solver in JCMsuite^66^, employing cylindrical rotational symmetry to retain full electromagnetic accuracy while minimizing computational costs. A Bayesian optimization algorithm was employed to simultaneously explore multiple structural degrees of freedom, including CBG-mesa diameter, ring thickness, ring gap, etching depth, and GaAs overgrowth thickness above the QD, each treated as a free parameter within fabrication-accessible bounds^67^ (refer to SI Table S1). Recent analysis has shown that even a single-ring CBG can deliver PEEs comparable to multi-ring architectures, while providing a substantially smaller device footprint and reduced fabrication complexity, which motivates our adoption of a single-ring design^68^. The SCQD was modeled as a point dipole emitter placed 135 nm above the buried-stressor layer, a height chosen to reflect the strain-driven vertical positioning of our SCQD growth. The oxidation aperture was fixed at 800 nm in diameter^61^, aligned with experimentally achievable values^58^. Among the optimized parameters, the GaAs overgrowth thickness plays a particularly critical role, influencing both the vertical cavity mode profile and practical growth feasibility. The algorithm autonomously converged toward geometries that ensured a maximal upward radiation into the desired NA cone and provided a direct blueprint for subsequent epitaxial and lithographic processes. The resulting optimized electric field distribution is shown in Fig. 1e (see SI section 3 for the high-resolution image).

To evaluate the robustness of this resonator design under realistic experimental conditions, we further performed scattering-mode simulations for PEE calculation in JCMsuite to quantify the impact of two key non-idealities: spectral mismatch and spatial misalignment of the emitter relative to the CBG resonator. In the first scenario, the emission wavelength of the dipole was swept from 900 to 960 nm, revealing the bandwidth tolerance of the optimized CBG and giving its compatibility with the natural inhomogeneity of QDs (see SI Fig. S3). In the second scenario, the dipole position was systematically offset laterally from the CBG-mesa center, reflecting the expected variability in QD nucleation even under site-controlled conditions. The quantitative implications of emitter-CBG misalignment are analyzed in detail in the subsequent section “Photon extraction efficiency”, where simulated offset-dependent PEEs are directly correlated with experimental measurements.

### Marker-free scalable integration of SCQDs into CBGs

The integration of SCQDs into CBG resonators was realized through EBL, employing a marker-free process that fully relies on the spatial determinism intrinsic to the buried-stressor growth technique. Unlike deterministic QD-cavity integration approaches that depend on resource-intensive imaging modalities such as CL or PL mapping, or on in-situ EBL, our method obviates the need for such procedures entirely. Notably, it also avoids the need for repeated cryogenic cooling cycles typically required for emitter localization prior to or during fabrication, further simplifying the overall process flow. Moreover, the CBG geometry is defined once for the target wavelength of 930 nm, and this design was implemented uniformly across the array, without emitter-specific wavelength-dependent mask modification. A 5 × 5 mm^2^ sample containing a 16 × 15 array of SCG-mesas was fabricated, from which a subset of 6 × 6 SCG-mesas was selected for CBG integration. Owing to the strain-induced control of QD nucleation, achieved through precise oxidation kinetics and strain localization, the QDs consistently form within ~100 nm-scale proximity to the SCG-mesa’s center^53^. Consequently, each CBG resonator was patterned without performing any individual alignment of the structure to the center of the SCG-mesa. Instead, the EBL process was initiated by navigating to a known SCG-mesa from the mask layout, performing standard write-field alignment and beam optimizations, and subsequently executing a matrix-based exposure using a predefined pitch between each mesa center, thus enabling automated and reproducible patterning across the full SCQD array. The scanning electron microscope (SEM) image in Fig. 1f showcases the remarkably high precision of the fabrication process. Representative structural characterization of the fabricated devices, including array overview, top-view, and side-view SEM images, is provided in the SI Fig. S5. Not only does our process simplify the workflow, but it also eliminates the need for alignment markers and auxiliary fabrication steps such as marker patterning via EBL and subsequent gold deposition. This streamlined fabrication flow, fully compatible with standard lithography techniques, enables scalable device realization across large-area samples. As such, the deterministic positioning of SCQDs and the simplicity of the integration protocol establish this approach as a compelling alternative to advanced image-based alignment techniques, offering a viable path for high-yield quantum photonic device fabrication even in facilities lacking sophisticated deterministic lithography infrastructure.

### Demonstration of scalability across an array of devices

**Fig. 2:**
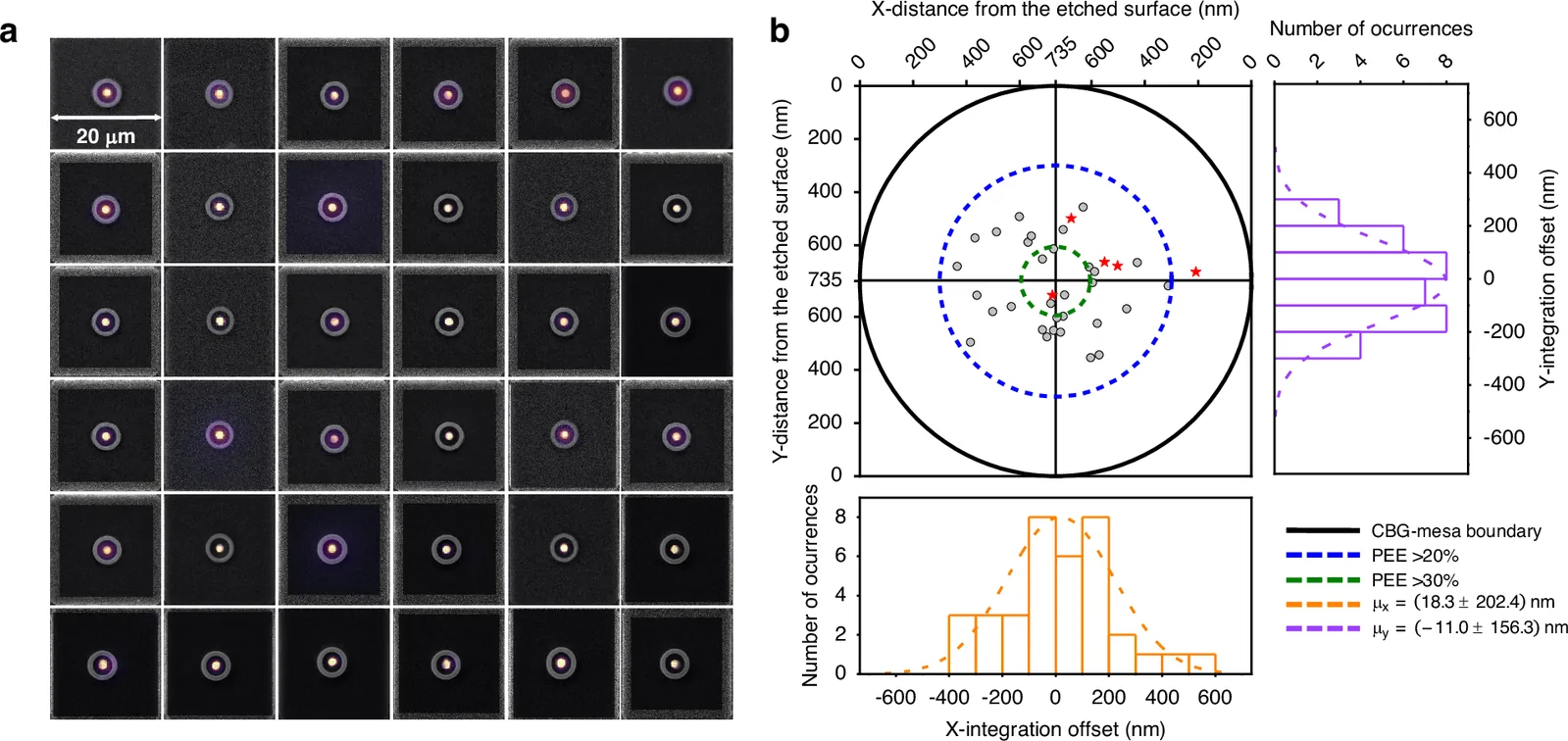
Demonstration of scalability across an array of devices. **a** Low-temperature (20 K) CL map (overlaid with SEM image) of a 6 × 6 SCQD-CBG device array (spectral range: (934 ± 6) nm), showing emission confined to the CBG-mesa centers with a 100% integration yield. **b** Statistical analysis of alignment accuracy from high-resolution CL scans (50 nm pixel size) of all 36 devices. QD positions were localized by 2D Gaussian fitting and compared with CBG-mesa centers extracted from SEM data. The resulting radial offset distribution, with mean displacements of *μ*_*x*_ = (18.3 ± 202.4) nm and *μ*_*y*_ = (− 11.0 ± 156.3) nm, confirms the high reproducibility of the SCQD-CBG alignment across the array and validates the robustness of our marker-free fabrication strategy

To assess the integration accuracy between the CBG resonators and the embedded SCQDs, we performed low-temperature CL mapping on a 6 × 6 array of SCQD-CBGs. Low-temperature (20 K) CL mapping of the full 6 × 6 SCQD-CBG array (Fig. 2a) reveals bright emission strictly confined to the CBG-mesa centers, confirming the precision of SCQD nucleation and their robust integration with the CBGs. Moreover, the emission wavelengths of all devices are spectrally homogeneous within (934 ± 6) nm, highlighting not only the reproducibility of the growth-fabrication scheme but also its scalability toward large, narrow-wavelength-range SPS arrays. Noteworthy, the reported spectral inhomogeneity lies in the range accessible by quantum confined Stark tuning^69,70^, which is compatible with the CBG concept^71,72^. The attainable Stark tuning range is governed predominantly by the underlying heterostructure and carrier confinement, rather than by the CBG geometry, and can therefore be engineered independently of the photonic design. The corresponding CL spectra for the device array shown in the CL maps are provided in the SI section 5 (Fig. S6).

#### Assessment of alignment accuracy

To quantify the alignment accuracy, high-resolution CL maps with 50 nm pixel size were analyzed within a statistical evaluation using a two-step method: a 2D Gaussian fit was applied to localize the QD emission within the CL data, while the CBG center was extracted from simultaneously acquired SEM images by identifying the CBG-mesa edges^68^. The resulting distribution of radial offsets provides a direct measure of the alignment accuracy, as shown in Fig. 2b, and yields mean displacements of *μ*_*x*_ = (18.3 ± 202.4) nm and *μ*_*y*_ = (−11.0 ± 156.3) nm. Applied across all 36 devices, this method revealed 100% fabrication yield in the sense that every SCQD was located within the central 1.47 μm-diameter CBG-mesa. In terms of optical performance, the yield can be further quantified based on the radial displacement criterion, where 5 devices exhibited a radial offset within ±125 nm, corresponding to a simulated PEE > 30%, and the remaining 30 devices fell within ±435 nm, yielding > 20% PEE. The present CBG design was optimized for PEE robustness and displacement tolerance rather than strong Purcell enhancement, and is therefore inherently more tolerant to emitter misalignment than hybrid CBG architectures. While deterministic alignment techniques based on QD pre-localization have achieved sub-50 nm accuracies in optimized workflows^21,68,73^, the results presented here are particularly significant as the entire integration process was carried out without the use of alignment markers, in-situ patterning, or prior emitter localization by CL or PL imaging. The observed alignment thus stems from a dual precision, intrinsic SCQD nucleation at the SCG-mesa center and photonic cavity placement registered to the same mesa center. The achieved integration accuracy highlights a practical and scalable pathway to realizing high-performance single-photon devices with reproducible efficiency, positioning this approach as a compelling alternative to deterministic alignment methods for large-scale quantum photonic architectures.

### Systematic characterization of SCQD-CBGs

A complete optical and quantum optical investigation was carried out on a selected subset of five SCQD-CBG devices from a 6 × 6 SCQD-CBG array, with systematically varied radial offsets between the SCQDs and the mesa center: SCQD-CBG1 (527 nm), SCQD-CBG2 (243 nm), SCQD-CBG3 (240 nm), SCQD-CBG4 (197 nm), and SCQD-CBG5 (54 nm) (indicated by red stars in Fig. 2b). All measurements were performed on the charged exciton (*X*^−^) emission line of each device, since it consistently appeared as the most dominant and intense transition. The charge states of these lines were assigned based on their systematic redshift relative to the neutral exciton, consistent with established results for negatively charged excitons in InGaAs QDs^74–76^. Comprehensive characterization, including *μ*PL, time-resolved lifetime measurements, second-order autocorrelation, HOM two-photon interference (TPI), as well as high-resolution linewidth determination via Fabry-Pérot interferometry (FPI), was conducted on all five devices, providing a complete assessment of their optical and quantum-optical properties and the impact of emitter radial displacement from the CBG-mesa center. A full description of the optical setup and measurement parameters, along with its schematic diagram, is provided in the SI (section 1). Details and raw datasets of all five SCQD-CBG devices are provided in the SI (section 6).

#### Optical characterization

**Fig. 3:**
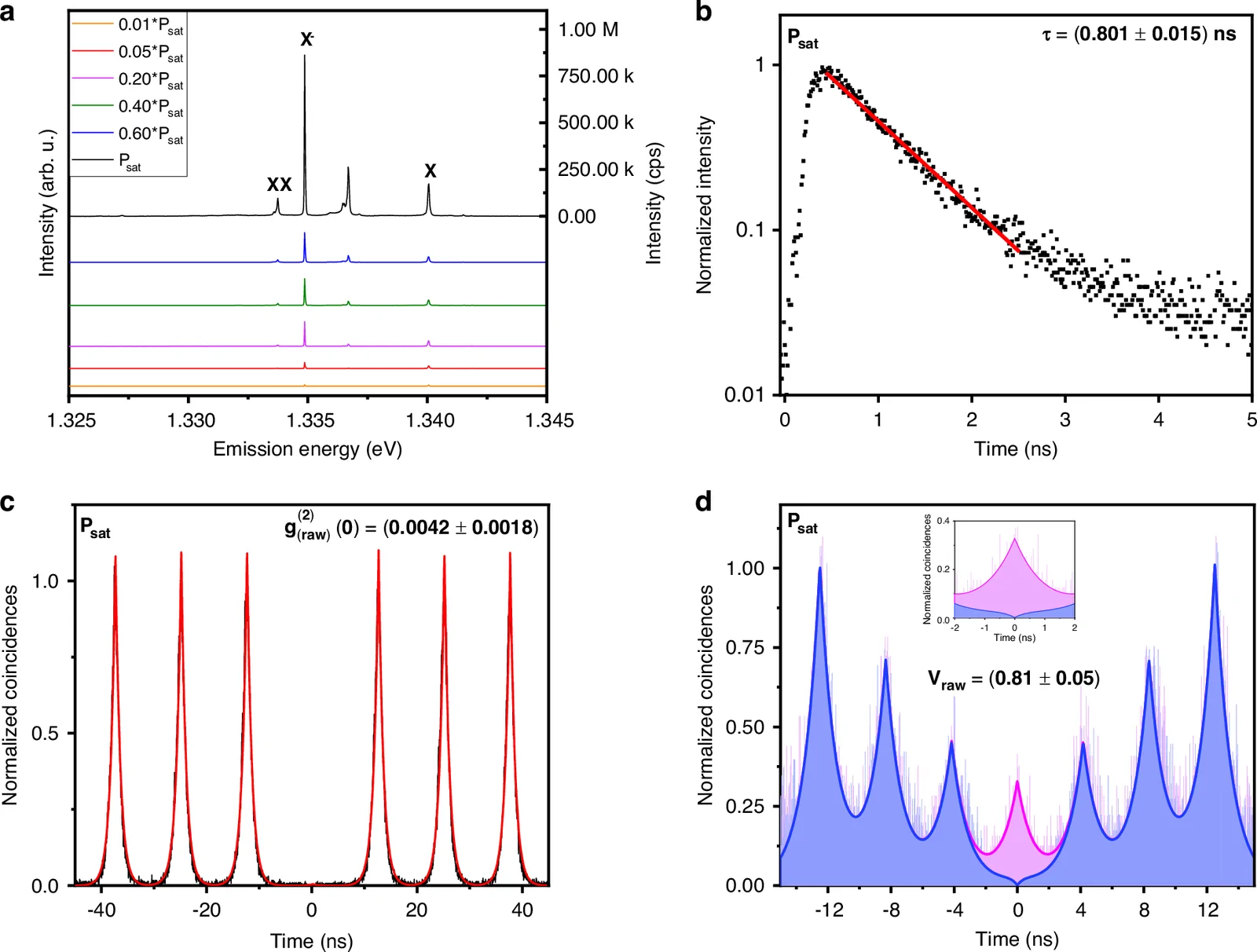
Optical and quantum optical characterization of the best-performing SCQD-CBG5 device (radial offset: 54 nm), measured at 4 K. **a** *μ*PL spectra under pulsed excitation at 870 nm for varying pump powers (vertically offset for clarity), revealing a max PEE of (47.1 ± 3.8)%. All subsequent measurements were performed under p-shell excitation at 911 nm and at the QD saturation power. **b** Time-resolved *μ*PL indicating a QD radiative decay lifetime of (0.80 ± 0.02) ns. **c** Second-order autocorrelation measurements demonstrating *g*^(2)^(0) = (0.0042 ± 0.0018), corresponding to a single-photon purity of (99.58 ± 0.18)%. **d** HOM TPI measurements exhibiting a raw visibility of (81 ± 5)%, evidencing very high photon indistinguishability

To evaluate the optical performance of the devices as a function of QD position, we conducted *μ*PL characterization on the five selected SCQD-CBG devices. Quasi-resonant excitation was performed using a tunable pulsed laser set to 870 nm. Corresponding power-dependent *μ*PL spectra Fig. 3a were recorded for the best-performing device (radial offset = 54 nm). This device, SCQD-CBG5, delivered the highest brightness with a maximum detected count rate of 2.73 MHz at saturation, corresponding to an end-to-end system efficiency of 3.4%. After correcting for the independently calibrated setup efficiency of (7.3 ± 0.3)% (see SI section 1), this corresponds to a PEE of (47.1 ± 3.8)%. To our knowledge, this is the highest value of PEE achieved for an SCQD integrated into a nanophotonic cavity, underscoring the effectiveness of our marker-free approach in combination with the CBG concept. This record value confirms that near-ideal alignment can be achieved without complex deterministic lithography or pick-and-place techniques, establishing a benchmark for scalable device fabrication. The raw *μ*PL spectra at saturation powers of all five SCQD-CBG devices are provided in the SI Fig. S8.

#### Polarization properties

To assess polarization anisotropy induced by lateral emitter displacement within the CBG-mesa, polarization-resolved measurements were evaluated for all five SCQD-CBG devices. All measurements were performed on the negatively charged exciton (*X*^−^), which does not exhibit fine-structure splitting. Therefore, any observed linear polarization originates from cavity-induced anisotropy rather than intrinsic excitonic splitting.

The degree of linear polarization (DLP) was calculated as:

1 $$DLP=\left(\frac{{I}_{max}-{I}_{min}}{{I}_{max}+{I}_{min}}\right)$$

where *I*_*max*_ and *I*_*min*_ denote the maximum and minimum integrated intensities extracted from Voigt fits to the polarization-resolved emission spectra. The extracted DLP values for the five devices (SCQD-CBG 5-1) are (1.51 ± 0.33)%, (1.29 ± 0.20)%, (7.57 ± 0.88)%, (5.36 ± 0.71)%, and (14.03 ± 1.11)%, respectively. A clear displacement-dependent trend is observed: near-centered emitters (<200 nm offset) exhibit very low polarization anisotropy (<2%), while larger radial displacements lead to progressively enhanced DLP. This behavior is consistent with the displacement-dependent symmetry-breaking trends predicted in ref. ^64^, although in our backside-DBR CBG design, the DLP remains comparatively less sensitive to lateral displacement. The device with the largest QD displacement (SCQD-CBG1 with a radial displacement of 527 nm) shows a DLP of 14%, consistent with pronounced symmetry breaking near the mesa boundary. These results confirm that the present CBG design preserves near-isotropic emission for well-aligned SCQDs while maintaining robust performance for moderate spatial offsets. The raw data underlying the DLP extraction are provided in SI Fig. S9.

#### Time-resolved PL studies

Time-resolved *μ*PL under quasi-resonant p-shell excitation was employed to evaluate the radiative lifetimes of the five selected SCQD-CBG devices, yielding values of (0.80 ± 0.02) ns, (0.70 ± 0.01) ns, (0.92 ± 0.01) ns, (1.01 ± 0.02) ns, and (1.02 ± 0.02) ns for SCQD-CBG5 through SCQD-CBG1, respectively. Fig. 3b displays the radiative decay lifetime of SCQD-CBG5. These numbers fall within the expected range for (buried-stressor) InGaAs QDs^60,77,78^ and show no systematic dependence on radial offset. As the CBGs were optimized for high PEE rather than Purcell enhancement, the absence of any systematic lifetime variation with radial displacement indicates that the radiative dynamics are governed predominantly by the intrinsic QD properties. This conclusion is corroborated by our FEM analysis (SI section 3), which yields a near-unity Purcell factor (between 0.9 and 1.3) over the SCQD emission band of (934 ± 6) nm, demonstrating that the CBG structure introduces no measurable spontaneous-emission enhancement. This also confirms that PEE can be engineered independently of lifetime modification, offering design freedom for future structures that may selectively target brightness or Purcell enhancement. Furthermore, the narrow lifetime spread across the devices suggests strong homogeneity of the buried-stressor QD ensemble, which is advantageous for reproducibility across large arrays. Time-resolved decay traces of the five SCQD-CBG devices are shown in the SI Fig. S10.

#### Single-photon emission purity

The single-photon nature of emission from the SCQD-CBG devices was rigorously assessed through second-order autocorrelation measurements using a Hanbury Brown and Twiss (HBT) setup. All measurements were performed at the respective saturation powers of the QDs, where multi-photon generation and background contributions are typically most pronounced. Excitation into the p-shell of the QD was employed to reduce carrier relaxation time jitter while avoiding laser stray light, thus allowing for clean isolation of the QD s-shell emission. The filtered emission was directed to a pair of SNSPDs, and coincidence histograms were acquired to extract the zero-delay second-order correlation *g*^(2)^(0), serving as a direct measure of the single-photon purity. Across the five devices (SCQD-CBG5-1), we observe consistently strong photon antibunching with *g*^(2)^(0) values of (0.4 ± 0.2)%, (0.6 ± 0.3)%, (2.6 ± 0.7)%, (2.3 ± 0.7)%, and (2.7 ± 0.3)%. Notably, the most spatially centered emitter exhibits a *g*^(2)^(0) = (0.0042 ± 0.0018), corresponding to a single-photon purity of (99.58 ± 0.18)%, highlighting the intrinsic suppression of multiphoton events even at QD saturation power (Fig. 3c). This value represents, to the best of our knowledge, the lowest multiphoton probability reported for any epitaxial SCQD system. Importantly, the measured *g*^(2)^(0) values exhibit no discernible dependence on emitter radial displacement from the CBG center, confirming that the suppression of multi-photon events is governed by intrinsic QD recombination dynamics rather than spatial coupling efficiency. The consistently sub-3% values across all structures affirm the robustness of the SCQD growth platform and the clean photonic environment of the CBG, both of which minimize spurious emission channels such as background luminescence or emission from adjacent states. Furthermore, no evidence of blinking is observed, as the emission remains stable during spectral acquisition and the measured *g*^(2)^(*τ*) exhibits no long-timescale bunching associated with charge-noise fluctuations. These results underline the scalability of our integration scheme, demonstrating high-purity single-photon generation without reliance on resonant pumping or spectral filtering, and establish the platform as a viable alternative to more complex deterministic or in-situ aligned systems. SI Fig. S11 presents the raw *g*^(2)^(*τ*) histograms for all five SCQD-CBG devices.

#### Photon extraction efficiency

**Fig. 4:**
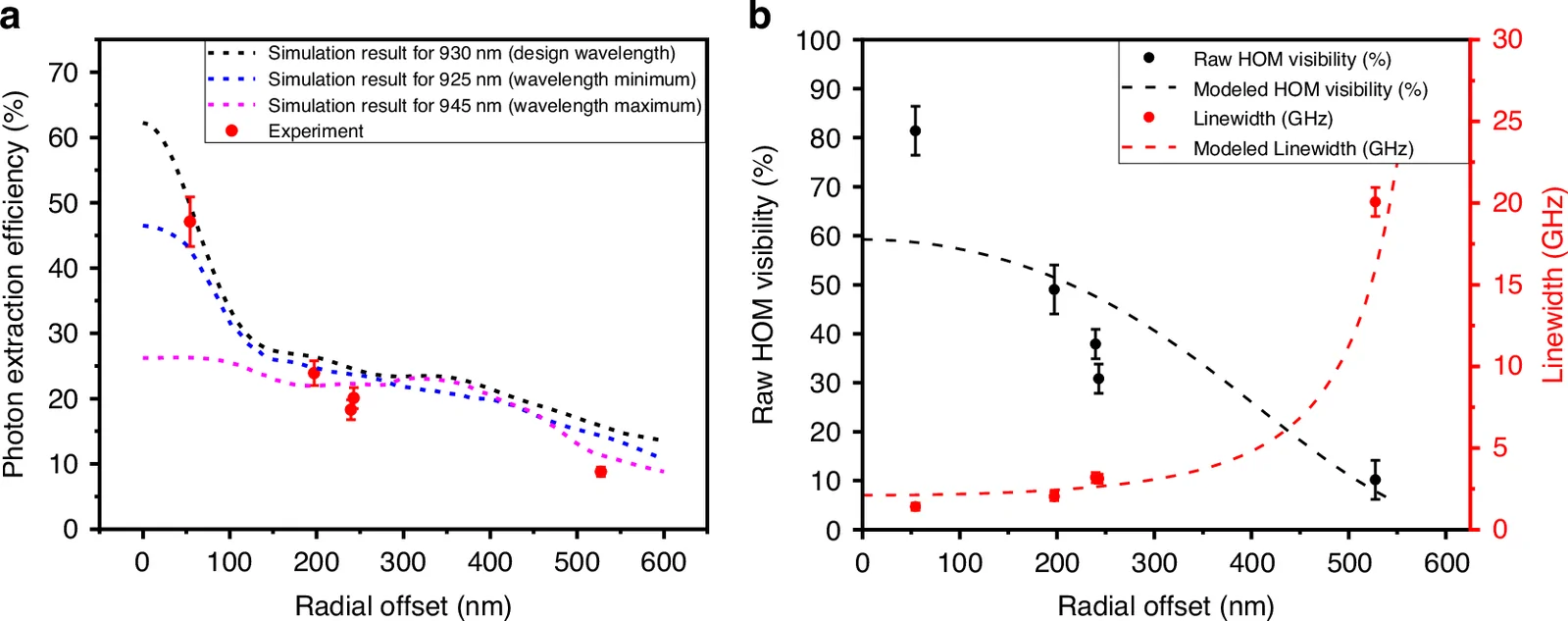
Assessment of optical and quantum optical device performance as a function of radial offset. **a** QD radial-offset dependence of PEE. FEM simulations are shown for emission wavelengths of 930 nm (design wavelength, black dashed line), 925 nm (blue dashed line), and 945 nm (magenta dashed line), with experimental results indicated by red circles. **b** Dependence of raw HOM-TPI visibility (%, left axis) and emission linewidth (GHz, right axis) on the QD radial offset. Experimental data are represented by black (visibility) and red (linewidth) circles, while the corresponding theoretical modeling is depicted by black and red dashed lines

To rigorously quantify the role of spatial alignment, we systematically correlated experimentally extracted PEEs from SCQD-CBG devices with varying radial offsets (red circles in Fig. 4a) to FEM simulations at 930 nm (black dashed line). The optimally aligned device, SCQD-CBG5 (54 nm radial offset) already delivers a PEE of (47.1 ± 3.8)%, in close agreement with FEM simulations predicting 50.2% at this displacement and a theoretical maximum of 62.2% for a perfectly centered emitter. Devices with larger radial offsets (SCQD-CBG4-1) exhibit reduced [simulated] efficiencies of (23.9 ± 1.9)% [26.4%], (18.3 ± 1.5)% [24.7%], (20.1 ± 1.6)% [24.6%], and (8.8 ± 0.7)% [15.9%], reflecting the expected decline in emitter-resonator coupling due to misalignment. Additional simulations at 925 nm and 945 nm (blue and magenta dashed lines), corresponding to the minimum and maximum emission wavelengths observed across our 16 × 15 SCQD array, were performed using identical structural parameters and varying only the dipole emission wavelength. These results show that while spectral sensitivity is pronounced for near-zero offsets, the PEE curves converge rapidly for radial displacements above ~150–200 nm. The overall experimental trend matches the simulated results, with minor deviations attributable to fabrication tolerances and local non-uniformities. This agreement between experiment and simulation establishes a quantitative benchmark for alignment tolerances in SCQD-CBG devices.

#### Linewidth characterization using Fabry-Pérot interferometry

Spectral linewidth measurements offer crucial insight into pure dephasing processes and play a central role in determining the suitability of QDs for SPS-based protocols. Under quasi-resonant p-shell excitation at saturation power, we employed a scanning FPI with a spectral resolution of 150 MHz to resolve the emission linewidths of the SCQD-CBG devices. Noteworthy, four out of five devices exhibited spectrometer resolution-limited (~30 μeV) linewidths, necessitating high-resolution FPI analysis. The most optimally aligned structure (SCQD-CBG5) demonstrated an emission linewidth of (1.41 ± 0.22) GHz, a remarkable result considering the complete absence of electrical or charge-noise suppression mechanisms, which are often used to reduce spectral jitter^24,79^. The measured linewidth of 1.41 GHz, though already remarkably narrow, remains above the Fourier-transform limit (~0.20 GHz for T1 = 0.8 ns), indicating residual spectral diffusion and excitation-induced dephasing under quasi-resonant pumping; strictly resonant (s-shell) excitation^80^ and charge stabilization in electrically contacted CBGs^81^ are expected to enable further approach toward the lifetime limit in future experiments. The other four devices yielded linewidths of (2.03 ± 0.25), (3.20 ± 0.31), and (3.12 ± 0.27) GHz, respectively. In contrast, the device with the largest radial displacement (527 nm), located ~200 nm away from the etched boundary of the CBG mesa, exhibited significant broadening to (20.07 ± 0.89) GHz, consistent with increased sensitivity to surface charge noise^82^. These findings establish a clear correlation between emitter-resonator alignment and spectral purity, reinforcing the importance of spatial precision in minimizing environmental noise and optimizing optical quality in integrating SPS.

Several findings ^82,83^ suggest that charge fluctuations caused by surface states at the etched surface of the CBG mesa play a major role in causing both decoherence and linewidth broadening. These fluctuating charges generate local electric fields that cause a time-varying energy shift in the emission frequency, leading to linewidth broadening. Since these fields decay with inverse distance squared, QDs with a higher radial displacement are affected more strongly by these fields, causing the aforementioned dependence of the linewidth on the displacement. As derived in the SI section 7, the linewidth broadening scales like

2 $${\gamma }_{{{\rm{deph}}}}(R)=c\,\frac{{R}_{0}^{2}+{R}^{2}}{{({R}_{0}^{2}-{R}^{2})}^{3}}$$

where *R* is the radial displacement, *R*_0_ is the radius of the CBG mesa, and *c* is a proportionality factor depending on the charge fluctuator density on the mesa interface. We stress that this relationship should be understood as describing the average behavior over many emitters, as it assumes a uniform distribution of surface charge states; in practice, the charge distribution can vary from sample to sample. Nevertheless, the theory curve in Fig. 4b agrees very well with the measured linewidths of the five samples, using *c* = 0.220 ns^−1^ μm^4^ as the only fitting parameter.

#### Photon indistinguishability

To directly assess the influence of spatial emitter placement on photon indistinguishability, we conducted HOM TPI measurements under pulsed quasi-resonant p-shell excitation, operating each SCQD at its saturation power. The 12.5 ns excitation pulses were converted into a sequence of double pulses separated by approximately 4.2 ns using a fiber delay line in the excitation path, and a matching 4.2 ns delay was introduced in one arm of the Mach-Zehnder interferometer to ensure temporal overlap of photons emitted following the two excitation events. The symmetric envelope modulation visible in the coincidence histograms arises from the convolution of the exponential radiative decay of the emitter with this double-pulse excitation sequence and the fixed interferometer delay. The raw HOM visibility is determined by integrating the coincidence histograms in the time interval of ±2 ns to obtain the total areas under the co- and cross-polarized curves, according to the formula:

3 $${V}_{HOM}=1-\left(\frac{{A}_{co}}{{A}_{cross}}\right)$$

where *A*_*co*_ and *A*_*cross*_ represent the total integrated areas of the co- and cross-polarized histograms, respectively. The coincidence histograms were acquired using fine temporal binning (40 ps) and accumulated until a predefined coincidence threshold was reached, ensuring statistically consistent visibility extraction while preserving the intrinsic interference dip profile. The results are striking that for the most precisely centered emitter (radially offset by only 54 nm), a raw HOM visibility of (81 ± 5)% (see Fig. 3d) is achieved, a record-breaking value for epitaxial SCQD systems and comparable to the best demonstrations using self-assembled QDs under p-shell excitation. For increasing lateral displacement we observe a clear degradation in indistinguishability, with visibilities dropping to (49 ± 5)%, (38 ± 3)%, (31 ± 3)%, and as low as (10 ± 4)% (the raw coincidence histograms from HOM TPI measurements on all five devices are included in SI Fig. S12.) in the most extreme case of SCQD-CBG1 with a lateral displacement of 527 nm. The latter corresponds to the emitter located ~200 nm away from the etched outer boundary of the CBG mesa, where decoherence mechanisms such as charge noise^81^ and surface-induced spectral fluctuations likely dominate in accordance with our theoretical description of the offset-dependent emission linewidth. To our knowledge, this is the first direct and quantitative demonstration of HOM TPI visibility as a function of emitter displacement within a photonic resonator, which provides a valuable basis for further device optimization. The ability to reach such high values of indistinguishability without complex tuning or charge suppression and using a SCG approach sets a new benchmark for integrated quantum emitters and opens up a clear path toward scalable quantum light sources for quantum technologies.

To obtain a better understanding of the underlying physics and limitations of the indistinguishability, we established a theoretical description based on a Markovian dephasing model. Within this approach, the HOM indistinguishability is expressed as ^84,85^

4 $${{\mathcal{I}}}=\frac{{\Gamma }_{{{\rm{rad}}}}}{{\Gamma }_{{{\rm{rad}}}}+{\gamma }_{{{\rm{deph}}}}}$$

To get a general trend as a function of emitter displacement, we evaluate Eq. (4) for the previously fitted linewidth *γ*_deph_(*R*) + *Γ*_rad_ depicted in Fig. 4b and a constant *Γ*_rad_ = 1.25 ns^−1^. In the same figure, we also show the indistinguishability that we obtain from our model. The theoretical values match well with the measurement results, especially at higher displacements, and capture the overall trend that indistinguishability improves with better center-positioning. The slight underestimation at small displacements results from the assumption of a uniform charge distribution that does not account for sample-specific variations in the local charge environment. In that sense, the results should be interpreted as describing an average behavior over many devices, rather than predictions for an individual sample. In addition, at this stage, we have neglected the effects of dielectric screening, meaning that charge-noise effects in proximity to the center should be overestimated in comparison to off-center positions, which might be another reason for the low HOM visibility predicted by this model. Regardless of the numerical deviations, the correct reproduction of the observed trends by the model calculations suggests the validity of the underlying assumption that charge noise mainly originates from the etched surface and can, consequently, be optimized by accurate emitter positioning. Notably, the calculations further reveal a saturation of the indistinguishability for *R* ≤ 50 nm, indicating that further improvements in spatial alignment are unlikely to significantly improve optical performance.

## Discussion

The results presented in this work establish a scalable and lithography-compatible route for integrating SCQDs into CBG resonators through a fully marker-free process. Built on a buried-stressor epitaxial platform and automated EBL patterning, the approach achieves sub-500 nm emitter-resonator alignment across an extended array, resulting in 100% process fabrication yield of working devices while maintaining displacement-dependent optical performance, with 5 devices operating in the >30% simulated PEE regime and the remaining 30 devices exceeding 20%. Correlative structural, spectroscopic, and quantum optical analyses reveal a clear correspondence between spatial offset and device performance, providing a unified experimental-numerical framework to quantify spatial-spectral coupling in quantum emitters.

Table 1 summarizes the performance metrics of five representative SCQD-CBG devices with increasing radial offset. The data confirm that emitter-resonator displacement primarily governs the optical coupling strength, directly impacting DLP, PEE, linewidth, and HOM TPI visibility, while intrinsic properties such as lifetime and single-photon purity remain largely unaffected. The best-performing device exhibits a PEE of (47.1 ± 3.8)%, single-photon purity of (99.58 ± 0.18)%, linewidth of (1.41 ± 0.22) GHz, and HOM visibility of (81 ± 5)%, setting a new benchmark for SCQD-based sources. These results demonstrate that epitaxial precision, combined with optimized resonator design, can yield high brightness and photon indistinguishability, both of which are essential for scalable quantum photonic architectures.

**Table 1:** Summary of performance metrics for five SCQD-CBG devices with varying radial offsets

Structure	Δ*R* (nm)	DLP (%)	PEE (%)	*L* (ns)	*g*^(2)^(0) (%)	LW (GHz)	*V* (%)
SCQD-CBG1	527	(14.03 ± 1.11)	(8.8 ± 0.7)	(1.02 ± 0.02)	(2.7 ± 0.3)	(20.07 ± 0.89)	(10 ± 4)
SCQD-CBG2	243	(5.36 ± 0.71)	(20.1 ± 1.6)	(1.01 ± 0.02)	(2.3 ± 0.7)	(3.12 ± 0.27)	(31 ± 3)
SCQD-CBG3	240	(7.57 ± 0.88)	(18.3 ± 1.5)	(0.92 ± 0.01)	(2.6 ± 0.7)	(3.20 ± 0.31)	(38 ± 3)
SCQD-CBG4	197	(1.29 ± 0.20)	(23.9 ± 1.9)	(0.70 ± 0.01)	(0.6 ± 0.3)	(2.03 ± 0.25)	(49 ± 5)
SCQD-CBG5	54	(1.51 ± 0.33)	(47.1 ± 3.8)	(0.80 ± 0.02)	(0.4 ± 0.2)	(1.41 ± 0.22)	(81 ± 5)

Δ*R* radial offset, *DLP* degree of linear polarization, *PEE* photon extraction efficiency, *L* lifetime, *LW* linewidth, *V* photon indistinguishability

A broader comparison with state-of-the-art alignment approaches is provided in Table 2. Despite eliminating alignment markers and an in-situ approach, the present platform achieves optical and quantum optical performance comparable to, and in several aspects exceeding, the results obtained using such complex schemes. In particular, the combination of high PEE, narrow linewidth, and high HOM visibility at QD saturation power under quasi-resonant excitation parallels that of marker-based SK and droplet-etched systems, while maintaining full compatibility with planar wafer-scale processing. The reproducibility and stability observed across multiple devices further underline the precision of the buried-stressor growth mode and the robustness of automated CBG patterning. We note that several landmark CBG demonstrations have reported photon indistinguishabilities exceeding 90%^86–88^. In contrast, the present work emphasizes fabrication scalability and systematic evaluation of displacement tolerance within a marker-free, site-controlled integration platform, rather than the pursuit of absolute record performance metrics.

**Table 2:** Overview of state-of-the-art QD alignment approaches and device performance metrics

QD	Structure	Δ*R* (nm)	IT	WL (nm)	PEE (%)	LW (*μ*eV)	*g*^(2)^(0)	*V* (%)	Ref
DE	Pillar+rings	50	MB	780	39	–	0.024	21	^73^
DE	Hybrid-CBG	66	iEBL	895	68	30	0.011	53	^68^
SK	Hybrid-CBG	32	MB	940	–	8	0.086	96	^21^
NH-SCQD	–	80	–	910	–	7	0.020	73	^96^
NW-SCQD	–	200	–	880	42	–	0.120	–	^97^
BS-SCQD	Microlens	–	iEBL	950	21	30	0.030	–	^61^
BS-SCQD	–	–	–	930	–	27	0.026	65	^57^
BS-SCQD	CBG	54	C	930	47	6	0.004	81	This work

Quantum optical measurements in refs. ^57,97^ were performed under continuous-wave (CW) excitation conditions

*QD* quantum dot growth method, Δ*R* radial offset, *IT* integration technique, *WL* wavelength, *PEE* photon extraction efficiency, *LW* linewidth, *V* photon indistinguishability, *Ref* reference, *DE* droplet etched quantum dots, *SK* Stranski-Krastanov grown quantum dots, *SCQD* site-controlled quantum dots, *NH* nanoholes, *NW* nanowires, *BS* buried-stressor quantum dot growth, *CBG* circular Bragg grating resonators, *MB* marker-based integration, *iEBL* in-situ electron beam lithography, *C* conventional EBL integration (not using alignment markers or in-situ techniques)

Together, these results establish a fabrication-efficient pathway toward scalable arrays of SPS with high indistinguishability. Looking ahead, the platform naturally extends toward wafer-scale arrays of SCQD-CBG devices, where Stark shift^69^, strain engineering^89^, temperature tuning^90^, or post-fabrication cavity trimming^91,92^ can spectrally tune distinct emitters to a common target resonance. Further improvements in growth-induced spectral homogeneity would correspondingly reduce the required post-fabrication tuning window, enhancing array-level scalability. Such fully tunable arrays open pathways to deterministic remote TPI between distinct emitters and to on-chip SPS with high indistinguishability for quantum networks^26,93^, as well as application-specific architectures such as neuromorphic photonic processors^94,95^. Ongoing work on electrically tunable SCQD arrays further supports the feasibility of deterministic spectral alignment for both remote and on-chip TPI in future device generations. The demonstrated alignment tolerance and fabrication robustness lay the groundwork for integrating large numbers of sources into complex photonic circuits, ultimately bridging the gap between proof-of-concept quantum light sources and large-scale, application-ready quantum photonic hardware.

## Methods

### Sample growth and nanoprocessing

The epitaxial structure was grown by MOCVD using AIX 200/4 (Aixtron AG). Growth is initiated at 700 ^∘^C with a 300 nm GaAs buffer layer on an n-type GaAs (001) substrate, followed by 33.5 Al_0.9_Ga_0.1_As/GaAs DBR mirror pairs and a 20 nm GaAs barrier layer. The buried-stressor layer comprises a 30 nm AlAs layer sandwiched between two 50 nm Al_0.9_Ga_0.1_As layers, capped by 80 nm of GaAs. This epitaxial template is patterned using UV-lithography into ~20 μm square SCG-mesas to expose the AlAs layer laterally and is subsequently transferred to a controlled wet thermal oxidation process known from standard industry-compatible VCSEL fabrication. Conducted at 420 ^∘^C in a water vapor/N_2_ atmosphere, the selective lateral oxidation of the AlAs layer converts its periphery into AlO_*x*_, leaving a central unoxidized AlAs aperture. The final aperture diameter is precisely defined by the lateral oxidation length, which is controlled via the SCG-mesa size in 100 nm increments, enabling precise tuning of the resulting tensile strain field. Real-time optical contrast monitoring ensures controlled termination of the oxidation to yield aperture openings between a few hundred nanometers and ~2 μm. To remove native oxides, the sample undergoes a surface treatment consisting of a 30 s dip in 75% H_2_SO_4_, followed by thorough rinsing in a deionized-water cascade until all acid residues are eliminated. The cleaned sample is then dried and immediately loaded into the MOCVD reactor for the subsequent overgrowth step. A 50 nm GaAs overgrowth layer is grown at 700 ^∘^C to planarize the surface, followed by the growth of ultralow-density (1 × 10^8^/cm^2^) In_0.33_Ga_0.67_As QDs at 500 ^∘^C, selectively nucleated at the center of the positions of maximum tensile strain^52^. A thin 0.7 nm GaAs capping layer, also grown at 500 ^∘^C, and a final 960 nm overgrowth layer, grown at 615 ^∘^C, complete the epitaxial structure.

Nanofabrication of the SCQD-CBGs was carried out by EBL using a Raith eLINE Plus system. The lithographic process relied exclusively on the predefined SCG-mesa as the positional reference and did not employ alignment markers or emitter localization. After navigating to a reference SCG-mesa defined in the layout, standard write-field alignment and beam optimization procedures were performed. CBG structures were patterned using an automated matrix-based exposure strategy with a fixed mesa-to-mesa pitch of 368 *μ*m, enabling reproducible patterning across the selected array without individual device alignment. Following exposure, the patterns were transferred into the semiconductor surface by dry etching, and residual resist was removed using standard cleaning procedures.

### Optical measurements

All optical experiments were performed at 4K using a closed-cycle cryostat equipped with high-stability nanopositioning stages. For excitation, a picosecond-pulsed optical parametric oscillator (OPO) operating at an 80 MHz repetition rate was used. Spectral analysis was carried out using a high-resolution (30-*μ*eV) grating spectrometer. The *μ*PL setup is extended to allow the emission from the sample to exit the monochromator via a flippable mirror towards a HBT setup, a HOM setup, a time-resolved measurement setup, or a portable FPI setup. A comprehensive description of the experimental setup is provided in SI section 1.

## Supplementary information

Supplementary Information for Scalable Quantum Photonic Platform Based on Site-Controlled Quantum Dots Coupled to Circular Bragg Grating Resonators

## Data Availability

The data that support the findings of this study are available from the corresponding authors upon reasonable request.
