# Supplementary material for: Scalable quantum photonic platform based on site-controlled quantum dots coupled to circular Bragg grating resonators
Source: Light Sci Appl. 2026 Jun 2;15:260. doi: 10.1038/s41377-026-02343-0 (PMC13230863; doi:10.1038/s41377-026-02343-0)
Supplement: Supplementary file 1 — Supplementary Information for Scalable Quantum Photonic Platform Based on Site-Controlled Quantum Dots Coupled to Circular Bragg Grating Resonators [file 41377_2026_2343_MOESM1_ESM.pdf]

Supplementary Information for Scalable Quantum Photonic Platform Based on Site-Controlled Quantum Dots Coupled to Circular Bragg Grating Resonators

Kartik Gaur[1*], Avijit Barua[1], Sarthak Tripathi[1], Léo J. Roche[1], Steffen Wilksen[2], Alexander Steinhoff[2], Sam Baraz[1], Neha Nitin[1], Chirag C. Palekar[1], Aris Koulas-Simos[1], Imad Limame[1], Priyabrata Mudi[1], Sven Rodt[1], Christopher Gies[2], Stephan Reitzenstein[1*]

[1]Institut für Physik und Astronomie, Technische Universität Berlin, Hardenbergstraße 36, Berlin, 10623, Germany.
[2]Institute for Physics, Carl von Ossietzky Universität Oldenburg, Oldenburg, 26129, Germany.

*Corresponding author(s). E-mail(s): kartik.gaur@tu-berlin.de; stephan.reitzenstein@physik.tu-berlin.de;

1 Experimental setup

All optical experiments were carried out at cryogenic temperatures (T = 4 K) in a closed-cycle cryostat equipped with high-stability nanopositioning stages. For broad pre-characterization of unpatterned SCG mesas, a CW laser diode at 660 nm was employed to excite the SCQDs at the mesa centers across the 5×5 mm^2 sample piece. A picosecond-pulsed optical parametric oscillator (OPO), operated at a repetition rate of 80 MHz, was tuned to the p-shell resonance of the QDs to ensure quasi-resonant excitation while minimizing excitation-induced dephasing and background fluorescence. The emitted PL was collected through a cold aspheric objective with an NA of 0.81, ensuring efficient collection of the upward-directed emission from the CBG structures. Stray laser light was effectively suppressed using a custom-tilted 950 nm long-pass filter, optimized for QD emission below 950 nm. The spectrally filtered signal was directed to a high-resolution grating spectrometer (1500 lines/mm), yielding a spectral resolution of $\sim$30 μeV, and subsequently imaged onto a nitrogen-cooled CCD camera for μPL measurements. For power-dependent and polarization-resolved spectroscopy, a computer-controlled variable neutral density filter wheel and a motorized half-wave plate combined with a polarizer enabled fine-tuned optical attenuation and polarization analysis.

For quantum optical and time-resolved measurements, the filtered emission was coupled into a polarization-maintaining single-mode fiber and routed to various fiber-based interferometric setups. Second-order correlation measurements were performed via a fiber-integrated Hanbury Brown and Twiss (HBT) configuration, comprising a 50:50 beam splitter and two superconducting nanowire single-photon detectors (SNSPDs) with a combined instrument response function (IRF) of 55 ps. Coincidence detection and correlation histograms were recorded using time-correlated single-photon counting (TCSPC) electronics. For two-photon interference measurements, Hong–Ou–Mandel (HOM) experiments were conducted in a fiber-based polarization interferometer. The OPO repetition rate was down-sampled to 4 ns to isolate individual photon wave packets. A calibrated polarization control stage consisting of quarter- and half-wave plates directed the signal into two optical delay arms, one of which included a 4 ns fiber delay. The two paths were recombined at a 50:50 fiber beam splitter and sent to two SNSPD channels for time-resolved coincidence analysis. High-resolution spectral linewidth measurements were performed using a scanning Fabry–Pérot interferometer (FPI) integrated into the detection path. The FPI, composed of a thermally and mechanically stabilized tunable etalon, featured a free spectral range (FSR) of 12.3 GHz and a resolution of $\sim$150 MHz. The interferometer was scanned at 5 mHz with a modulation amplitude of 500 mV$_{p-p}$, and the transmission signal was coupled into a multimode fiber and detected by a single-photon avalanche diode (SPAD) channel. A schematic representation of the full experimental setup is shown in Fig. S 1

The setup efficiency was determined by calibrating the overall detection efficiency of the μPL system using a Toptica DL 100 CW laser tuned to the QD emission wavelength ($\sim$ 930 nm). This yielded a system efficiency of $(7.3 \pm 0.3)\%$, accounting for cumulative losses along the collection path, including transmission through optical elements and the CCD's quantum efficiency at the corresponding wavelength [1].

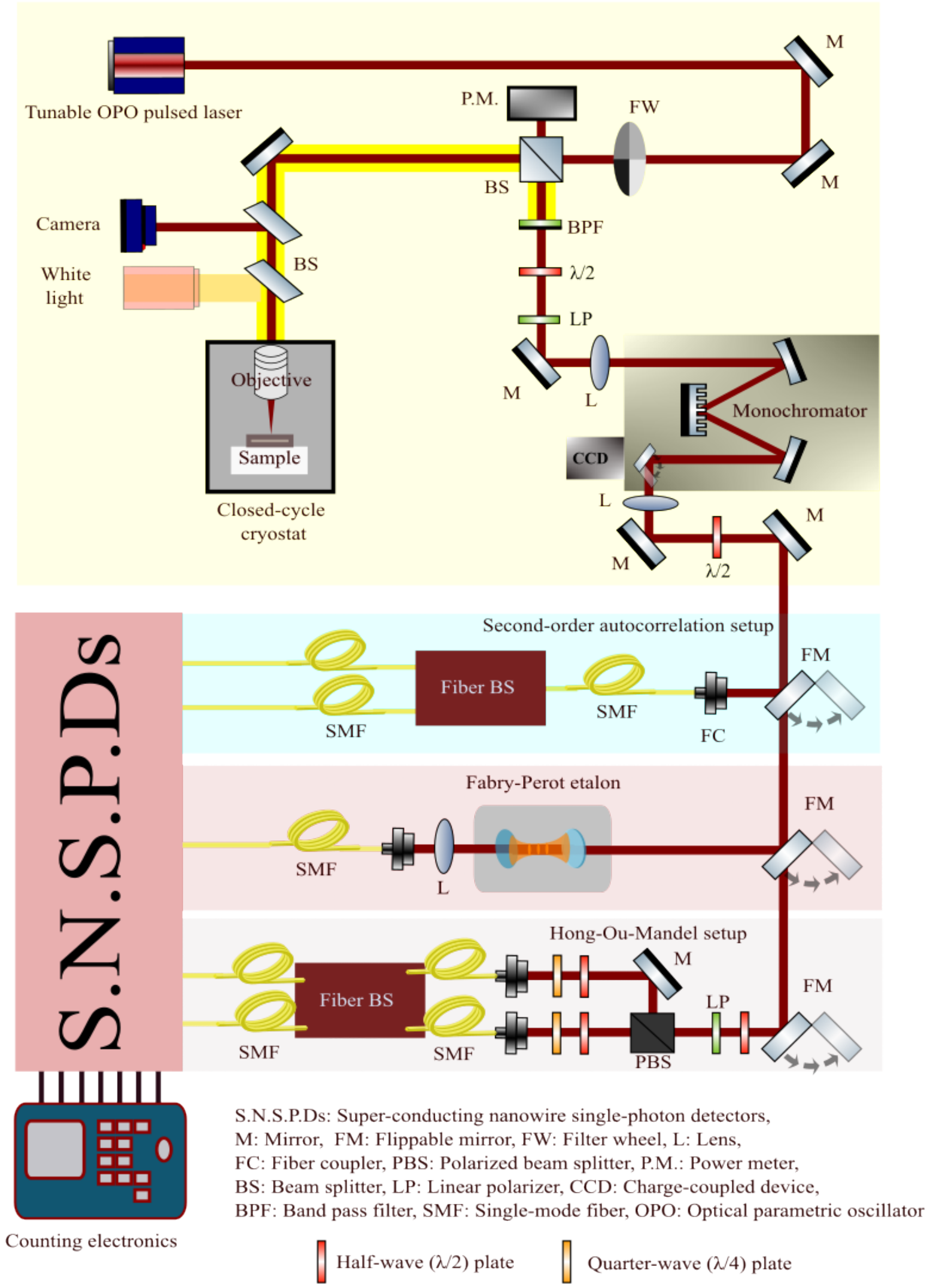

Fig. S 1 Schematic representation of the experimental setup.

2 Optical assessment of SCG-mesas

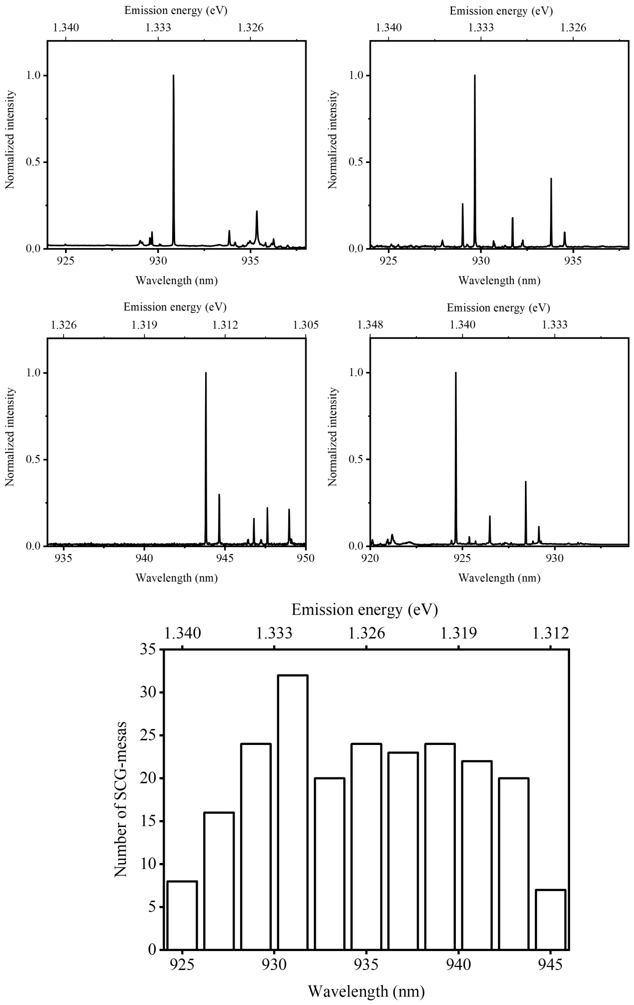

Fig. S 2 Representative μPL spectra of SCG-mesas, with the accompanying histogram showing the emission energy distribution across over 200 individual SCG-mesas.

Optical characterization of the SCG-mesas was performed in above-band excitation μPL experiments using a CW diode laser at 660 nm. The excitation beam was focused onto the center of each mesa to probe the embedded SCQDs. Emission was collected and analyzed to extract key statistics, including the emission energy distribution, across the 5×5 mm^2 sample piece. Exemplary emission spectra for individual SCG-mesas are shown (see Fig. S 2), accompanied by a histogram summarizing the emission energies of the dominant line from 220 SCG-mesas, providing a quantitative assessment of sample uniformity and spectral reproducibility.

3 Design and simulation of CBG resonators

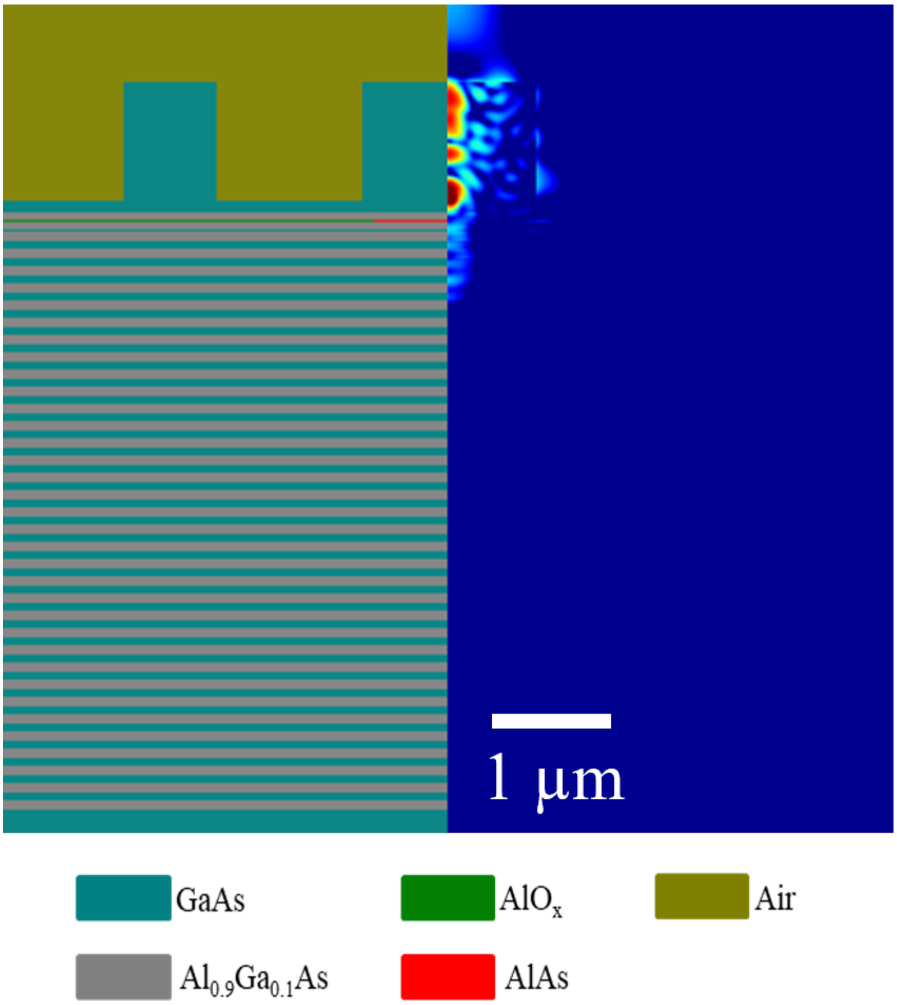

Fig. S 3 Finite element method (FEM) simulations showcasing the optimized SCQD-CBG geometry (left) and the corresponding electric-field distribution (right).

In Fig. S 3, the left panel shows the simulated cross-section of the optimized SCQD-CBG structure, while the right panel displays the corresponding FEM-calculated electric-field distribution [2]. The simulated electric field distribution shows that the emission originates at the SCQD location and is efficiently redirected upward. The DBR beneath the cavity suppresses downward leakage, leading to a vertically collimated radiation pattern consistent with the high PEE predicted for the optimized

Table S 1 Optimized structural parameters of the fabricated SCQD-CBG devices as used in FEM simulations.

**Parameter**	**Value (nm)**
Mesa diameter	1470
Ring thickness	740
Ring gap	1150
Etching depth	980
Oxidation aperture	800

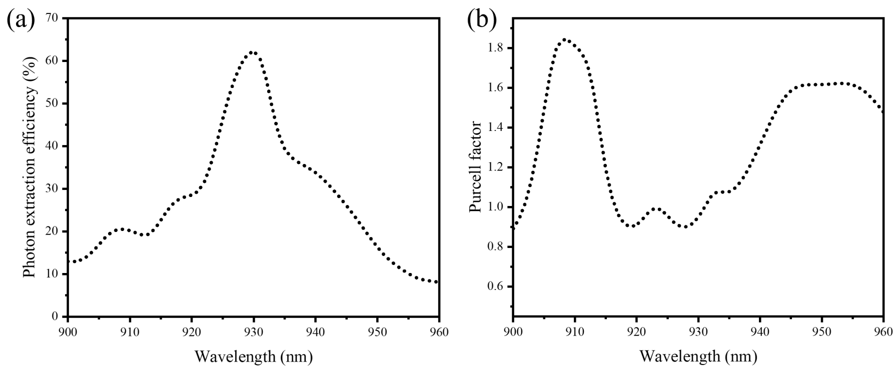

Fig. S 4 FEM simulation results of the (a) PEE and (b) Purcell factor of the optimized CBG design as a function of dipole emission wavelength.

design. The final set of optimized structural parameters from FEM simulations, including mesa diameter, etch depth, ring thickness, ring gap, and oxidation aperture, is summarized in Table S 1. For these design parameters, a spectral sweep of the dipole emission wavelength (from 900 to 960 nm) was performed, revealing a maximum PEE of 62.2% at the target resonance of 930 nm (Fig. S 4 (a)), which defines the operational bandwidth and informs tolerance to QD spectral inhomogeneity [3]. In the same simulation framework, we also evaluated the expected Purcell enhancement (Fig. S 4 (b)). Because the structure was optimized solely for maximizing PEE, the simulated Purcell factor remains very close to unity across the relevant spectral window. In particular, within the emission range of our SCQD-CBGs ((934 ± 6) nm), the Purcell factor varies only between 0.9 and 1.3. This shallow variation indicates that the CBG introduces no significant modification of the radiative rate, and therefore, no measurable lifetime shortening is expected in the experiments [4]. The experimentally observed lifetimes can thus be interpreted as intrinsic emitter properties, unaffected by cavity-induced enhancement. To further quantify the confinement limits of the present SCQD-CBG geometry, we performed additional FEM simulations exploring the trade-off between PEE and Purcell enhancement. When constraining the design to

maintain PEE >= 50%, the maximum achievable Purcell factor is approximately 1.6 (with $\sim$56% simulated PEE). Relaxing the PEE constraint allows Purcell factors up to $\sim$2.4, with a corresponding PEE of $\sim$44%. In the present work, the etching depth was intentionally limited to preserve structural robustness and compatibility with the stressor-based layer stack. However, if more complex fabrication strategies enabling significantly deeper etching ($\sim$1600 nm) are employed, the same geometry can, in principle, achieve a Purcell factor of $\sim$10, albeit with a reduced extraction efficiency of $\sim$21%. The moderate Purcell enhancement observed experimentally, therefore, reflects an intentional design trade-off rather than a fundamental limitation of the platform.

4 Structural characterization of the SCQD-CBG devices

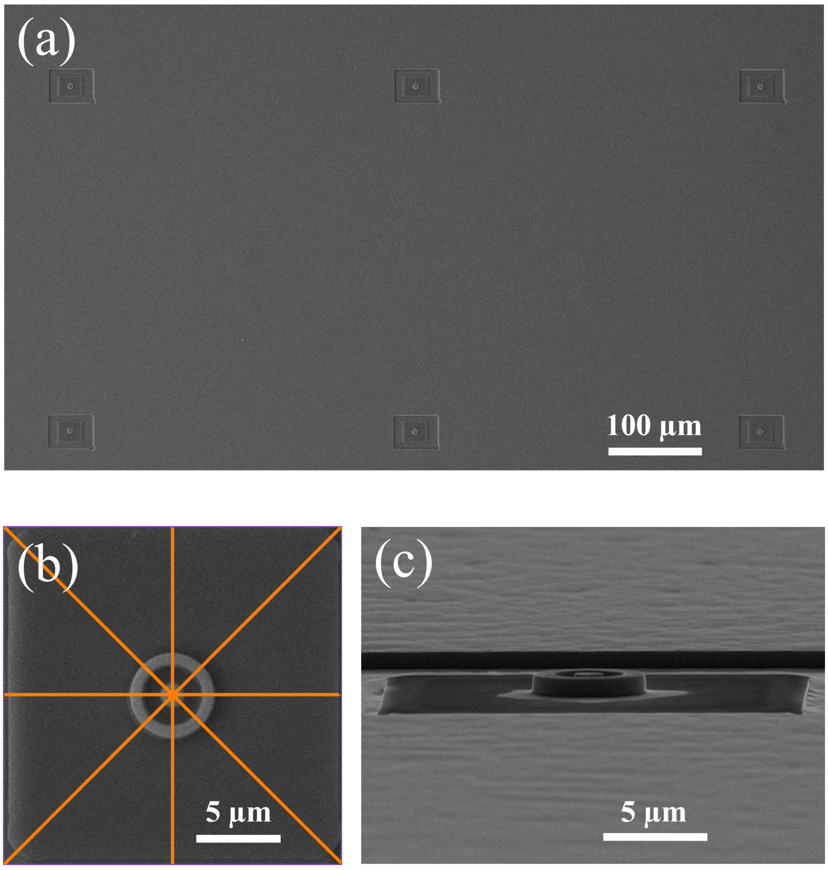

Fig. S 5 Structural characterization of SCQD-CBG devices via SEM. (a) Low-magnification SEM image of a representative 2 × 3 section of the fabricated device array. (b) High-resolution top-view SEM image of a single device. The orange cross-hair lines indicate the geometric center of the CBG structure, illustrating the emitter alignment of the SCQD-CBG device relative to the SCG-mesa. (c) Side-view SEM image of the device, showing the etching profile.

This section presents representative SEM images of the fabricated SCQD-CBG devices, emphasizing structural quality and lithographic precision. In Fig. S 5, plan-view images illustrate CBG geometry together with array scalability, while the side-view image reveals the etched SCQD-CBG profile and well-defined sidewall structure.

5 Cathodoluminescence emission spectra of the SCQD-CBGs

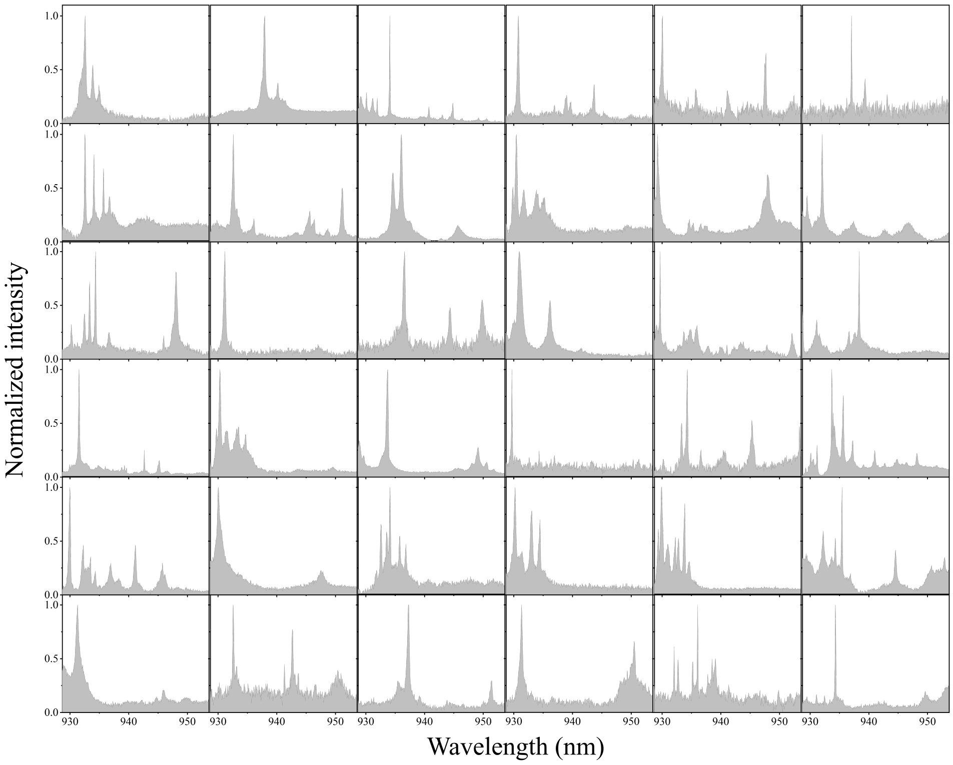

Fig. S 6 Low-temperature CL spectra corresponding to the 6 × 6 array of SCQD-CBG devices shown in the main text. Each panel displays the emission spectrum acquired from the center of the respective CBG, complementing the CL intensity maps.

As discussed in the main text, low-temperature CL mapping was performed across the complete 6×6 array of SCQD-CBG devices to assess spatial alignment and spectral reproducibility. Each device exhibits a localized emission spot at the mesa center, confirming precise positioning of the CBG-mesa with respect to the SCQDs. The corresponding CL spectra from the CBG-mesa centers are shown in Fig. S 6. Fig. S 7 shows the histogram of the dominant emission wavelength obtained from the CL spectra shown above, revealing a narrow spectral spread that reflects the high uniformity of the SCQD emission across the array.

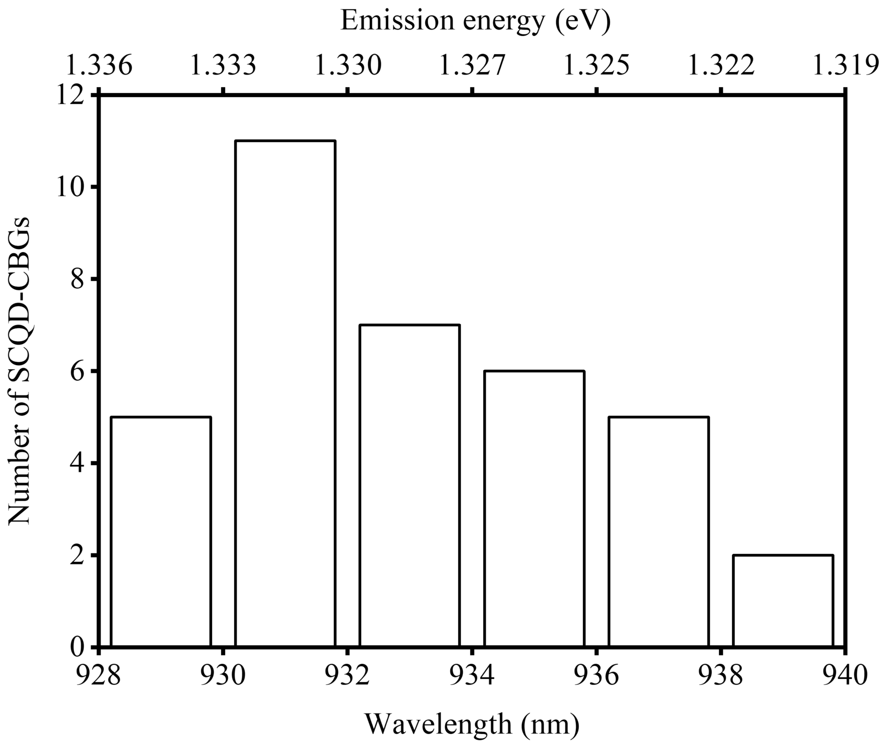

Fig. S 7 Statistical distribution of the dominant QD emission wavelength extracted from the CL spectra across the 6×6 device array.

6 Comparison of devices with varying radial offsets

To assess the influence of the QD-resonator alignment, we investigated five CBGs with different spatial offsets between the emitter position and the mesa center: SC-CBG1 (527 nm), SC-CBG2 (243 nm), SC-CBG3 (240 nm), SC-CBG4 (197 nm), and SC-CBG5 (54 nm), as discussed in the main text. For each device (SC-CBG 1-5), we provide the raw experimental data corresponding to the emission spectra, decay lifetimes, second-order autocorrelation, and HOM TPI. These datasets complement the summarized values presented in the main text.

6.1 Emission Spectra

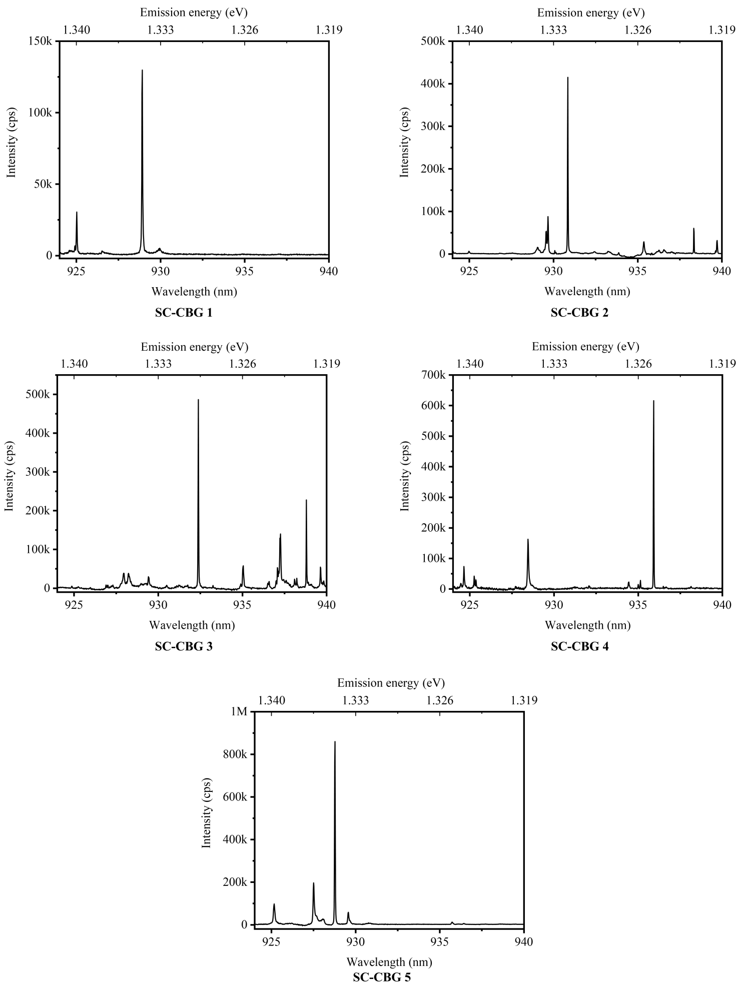

Fig. S 8 μPL spectra of the five devices (SC-CBG 1-5) with different radial offsets.

The μPL spectra of the five devices are displayed in Fig. S 8. The spectra confirm single QD emission, with variation in intensity reflecting differences in PEE due to the offset.

6.2 Degree of linear polarization

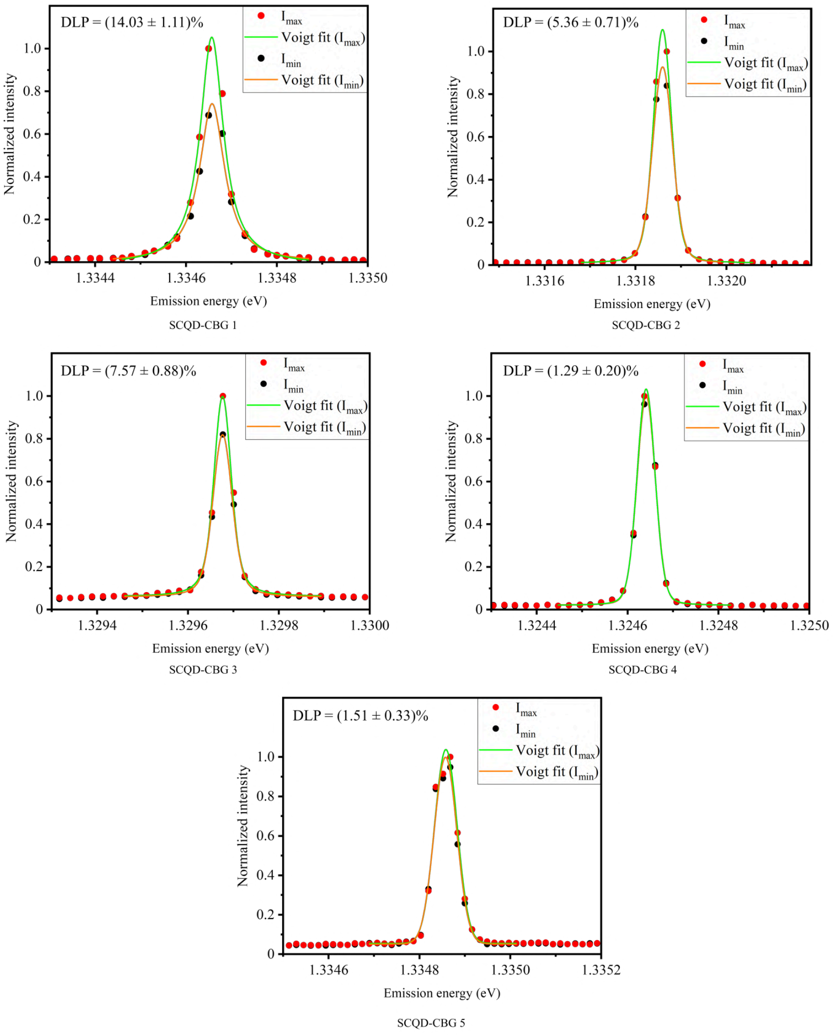

Fig. S 9 Polarization-resolved emission spectra of the negatively charged exciton (X^-) for SCQD-CBG 1-5 with varying radial offsets. Red and black scattered points correspond to the maximum (I_{max}) and minimum (I_{min}) intensities from the polarization-resolved measurements, respectively. Green and orange lines represent the corresponding Voigt fits to I_{max} and I_{min}. The extracted DLP values are indicated in each panel.

Fig. S 9 presents the degree of linear polarization (DLP) analysis for the five SCQD-CBG devices. The extracted DLP values range from $(1.29\pm0.20)\%$ to $(14.03\pm1.11)\%$. Devices with minimal radial displacement exhibit near-isotropic emission, whereas increasing offsets lead to enhanced polarization anisotropy. This behavior is consistent with cavity-mode symmetry breaking induced by lateral emitter displacement [5].

6.3 Lifetimes

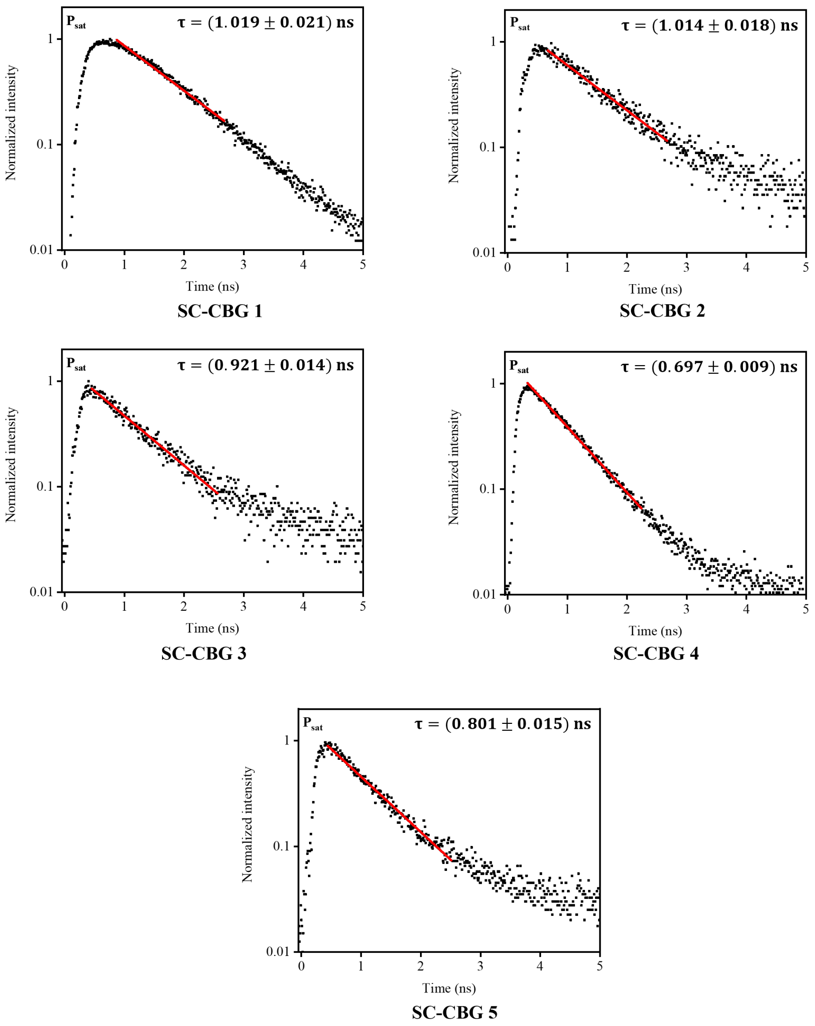

Fig. S 10 Time-resolved μPL decay curves of the five devices (SC-CBG 1-5) with different radial offsets.

Fig. S 10 shows the time-resolved μPL plots for the five SCQD-CBG devices. Exponential fits to the decay yield radiative lifetimes in the range 0.697 ns to 1.019 ns. Importantly, the lifetimes exhibit no systematic dependence on radial offset; the device-to-device spread is comparable to the intrinsic variability of (buried-stressor) InGaAs QDs. The absence of a clear offset-dependent trend indicates a negligible modification of the radiative decay by the CBG structures, consistent with a device design that prioritizes PEE over Purcell enhancement.

6.4 Second-order autocorrelation

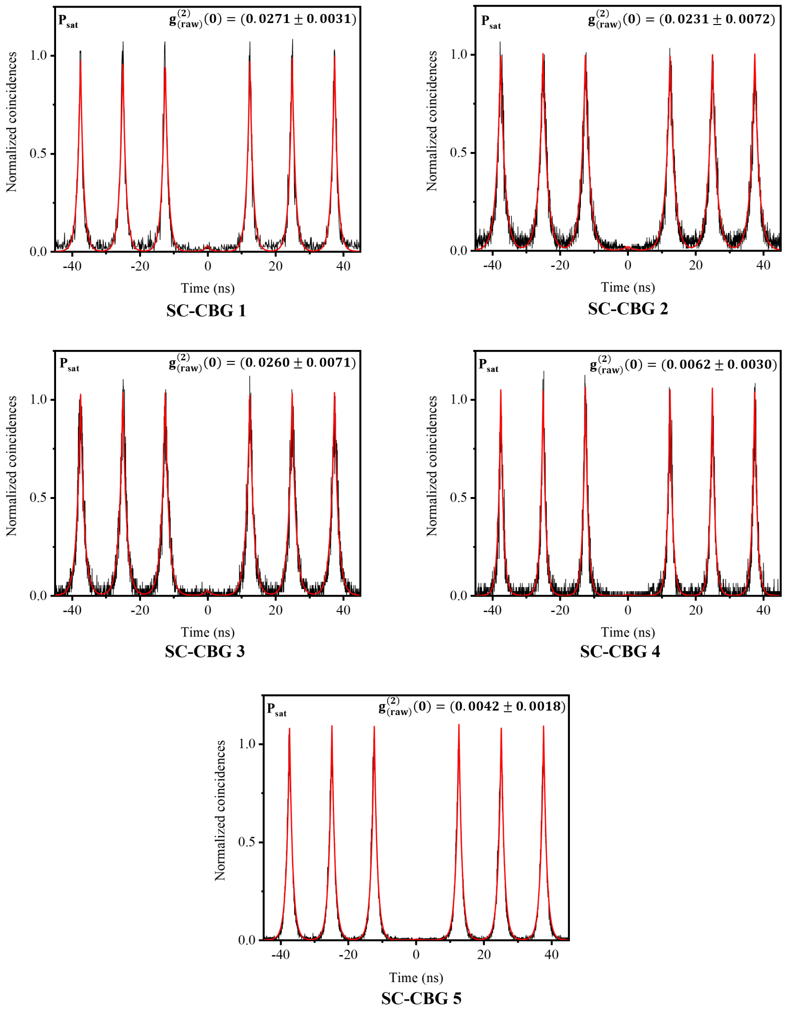

Fig. S 11 Second-order autocorrelation $g^{(2)}(\tau)$ histograms of the five devices (SC-CBG 1-5) with different radial offsets, all exhibiting pronounced antibunching at zero delay.

The raw (and fitted) second-order autocorrelation histograms, $g^{(2)}(\tau)$, for the five devices are shown in Fig. S 11. All devices exhibit a pronounced antibunching dip at zero delay, confirming pure single-photon emission. The extracted $g^{(2)}(0)$ values span a narrow range across the different offsets, with variations attributable to intrinsic emitter properties. Importantly, no systematic dependence on radial displacement is observed, consistent with the expectation that the single-photon purity is governed primarily by the QD itself rather than the CBG alignment.

6.5 Hong-Ou-Mandel two-photon interference

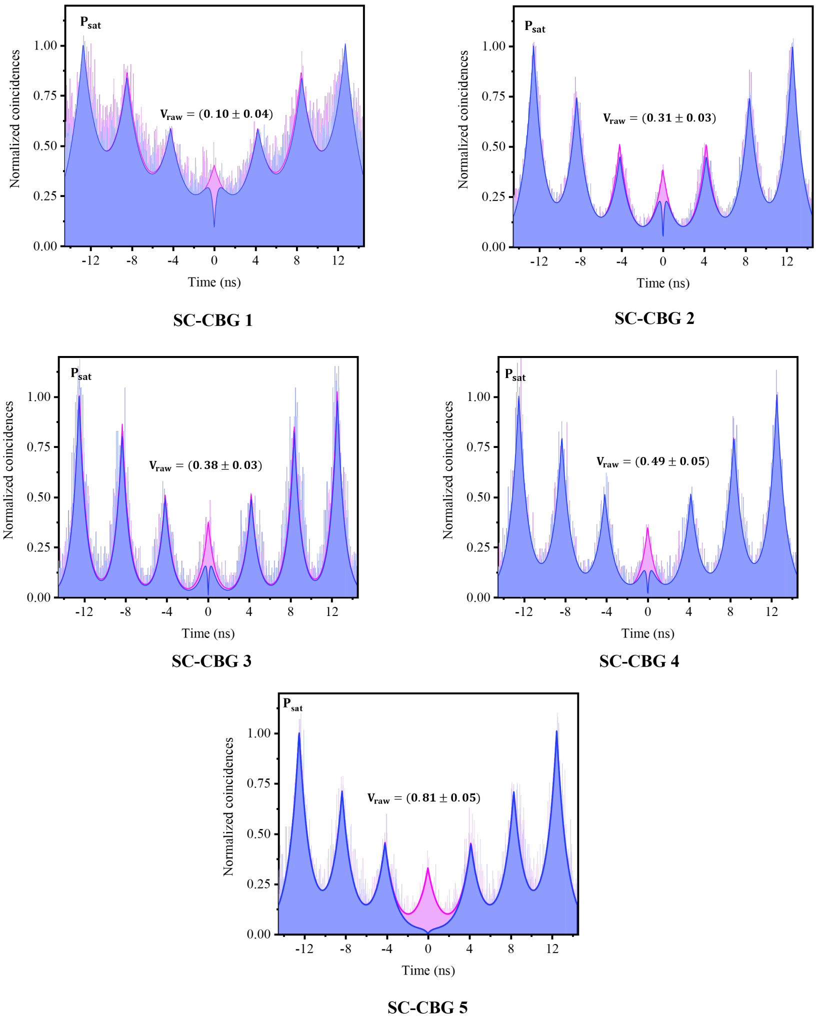

Fig. S 12 HOM TPI measurements for the five devices (SC-CBG 1-5) with different radial offsets, illustrating offset-dependent variations in photon indistinguishability.

Fig. S 12 shows the raw (and fitted) HOM TPI histograms for the five SCQD-CBG devices. The extracted HOM visibilities reveal a clear dependence on QD-resonator alignment, with higher indistinguishability for well-centered emitters and a systematic reduction as the radial offset increases. These data provide a direct experimental view of how emitter positioning affects TPI across devices.

7 Modeling of charge noise amplitude

To get an estimate of the charge noise amplitude, we assume that the most dominant contributions to the charge noise stem from surface states at the CBG boundary [6, 7]. At each surface charge trap, a fluctuating charge $q_i(t)$ is located, inducing an electric field $F_i(\vec{r}, t)$. The total electric field at the QD position $\vec{R}$ is then given by

$$F(\vec{R}, t) = \sum_i F_i(\vec{R}, t) = \sum_i \frac{1}{4\pi\epsilon_0\epsilon_\mathrm{r}} \frac{q_i(t)}{|\vec{R} - \vec{r}_i|^2} \tag{1}$$

This electric field will cause an energy shift of

$$\Delta E(\vec{R}, t) = \sum_i \frac{1}{4\pi\epsilon_0\epsilon_\mathrm{r}} \frac{q_i(t)}{|\vec{R} - \vec{r}_i|^2} d_i \tag{2}$$

with d_i being the projection of the dipole moment onto the direction of the electric field. This permanent dipole moment is caused by the average charge environment. The spectral noise amplitude at the QD location is given by

$$S(\omega) = \int \mathrm{d}t \mathrm{e}^{\mathrm{i}\omega t} \langle \Delta E(\vec{R}, t) \Delta E(\vec{R}, 0) \rangle \propto \sum_{ij} \int \mathrm{d}t \, \mathrm{e}^{\mathrm{i}\omega t} \langle q_i(t) q_j(0) \rangle \frac{d_i d_j}{|\vec{R} - \vec{r}_i|^2 |\vec{R} - \vec{r}_j|^2} \tag{3}$$

The fluctuations at different sites are assumed to be independent, which leads to vanishing cross-correlations for $i \neq j$. We define $f_i = \int \mathrm{d}t \mathrm{e}^{\mathrm{i}\omega_0 t} d_i^2 \langle q_i(t) q_i(0) \rangle$ as the weighted noise spectrum of a single fluctuator at a fixed frequency ω_0. The noise amplitude at this frequency is then given by

$$S := S(\omega_0) \propto \sum_i f_i(\omega) \frac{1}{|\vec{R} - \vec{r}_i|^4} = \sum_i \frac{f_i(\omega)}{(R^2 + R_0^2 - 2RR_0 \cos\varphi_i)^2} \tag{4}$$

Here, φ_i is the relative angle as defined in Fig. S 13, R is the radial displacement of the QD, and R_0 is the radius of the CBG. Introducing a fluctuator density $\rho(\varphi) = \sum_i f_i \delta(\varphi - \varphi_i)$, we can rewrite this as an integral:

$$S \propto \int_0^{2\pi} \frac{\mathrm{d}\varphi \, \rho(\varphi)}{(R^2 + R_0^2 - 2RR_0 \cos\varphi)^2} \tag{5}$$

In general, the fluctuator density will depend on the sample and is unknown. To get an estimate for the average trend of the amplitude of the charge noise in a random sample, we assume it to be constant, since, on average, there is no angle preference. Then, we write the average noise amplitude as

$$S \propto \int_0^{2\pi} \frac{\mathrm{d}\varphi}{(R^2 + R_0^2 - 2RR_0 \cos\varphi)^2} = 2\pi \frac{R_0^2 + R^2}{(R_0^2 - R^2)^3} \tag{6}$$

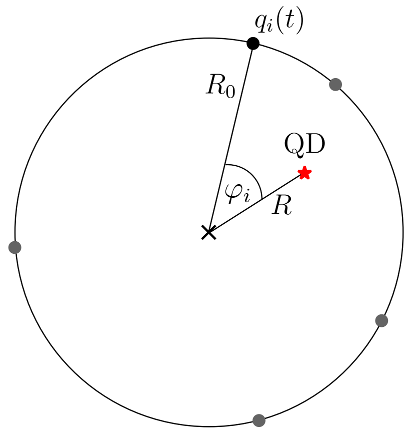

Fig. S 13 Sketch of the model underlying the calculation of the charge noise amplitude. The quantum dot with radial offset R is displayed in red, while the charge fluctuators are randomly distributed on the etched surface of the CBG and displayed as gray dots. For one fluctuator, the definition of angle φ_i is shown.

For a single sample, this assumption is unjustified, but we use it here to get a general trend of the average charge noise amplitude as a function of the radial displacement. The dephasing rate γ_{deph} due to charge fluctuations will be proportional to the noise amplitude:

$$\gamma_{\mathrm{deph}}(R) = c\frac{R_0^2 + R^2}{(R_0^2 - R^2)^3} \tag{7}$$

Together with the radiative recombination rate $\Gamma_{\mathrm{rad}} = T_1^{-1}$, we can calculate the Lorentzian linewidth as

$$\kappa = \Gamma_{\mathrm{rad}} + \gamma_{\mathrm{deph}} \tag{8}$$

and determine the proportionality constant c by fitting to the measured linewidth. From this we get a proportionality factor of $c = 0.220\,\mathrm{ns}^{-1}\mathrm{\mu m}^4$. The Hong-Ou-Mandel indistinguishability can be calculated using

$$\mathcal{I} = \frac{\int_0^\infty \mathrm{d}t \int_0^\infty \mathrm{d}\tau \, |C(t,\tau)|^2}{\int_0^\infty \mathrm{d}t \int_0^\infty \mathrm{d}\tau \, C(t,0)C(t+\tau,0)} \tag{9}$$

where $C(t,\tau)$ is the two-time correlation function of the optical transition. For a two-level system with Markovian dephasing, this correlation function is given by

$$C(t,\tau) = \mathrm{e}^{-\Gamma_{\mathrm{rad}}t - (\Gamma_{\mathrm{rad}} + \gamma_{\mathrm{deph}})\tau/2} \tag{10}$$

resulting in an indistinguishability of $\mathcal{I} = \frac{\Gamma_{\mathrm{rad}}}{\Gamma_{\mathrm{rad}} + \gamma_{\mathrm{deph}}}$. For the computational results plotted in Fig. 5 in the main text, we chose a constant recombination rate $\Gamma_{\mathrm{rad}} =$

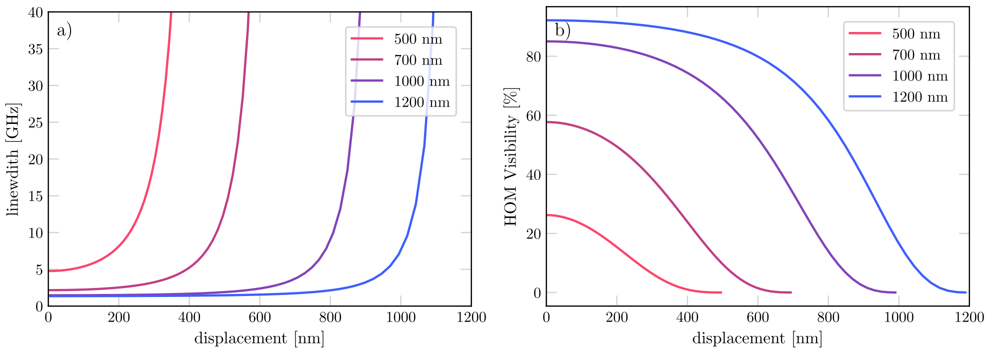

Fig. S 14 Theoretically calculated linewidth (a) and HOM visibility (b) as a function of the radial displacements for different mesa radii.

$1.25\,\mathrm{ns}^{-1}$ to better showcase the general trend without being restricted to only five data points where the lifetime has been measured. Fig. S 14 shows both the linewidth and HOM visibility calculated for different mesa radii R_0. As expected, a larger mesa diameter leads to a lower linewidth and higher visibility for equal alignment accuracies. Also, the saturation displacement, i.e., the displacement range in which better alignment only marginally increases the visibility, is larger for larger mesas. If one considers a mesa with radius $R_0 = 1200\,\mathrm{nm}$, an improvement in alignment accuracy only slightly improves the HOM visibility. It needs to be pointed out that these results assume the same fitting constant c (which includes the charge trap density on the surface) and radiative lifetime for all radii, which might vary with mesa diameter.

References

[1] Barua, A. *et al.* Deterministic nanofabrication of quantum dot-circular Bragg grating resonators with high process yield using in situ electron beam lithography. *Advanced Quantum Technologies* **9**, e00782, doi: 10.1002/qute.202500782 (2026).

[2] JCMwave. JCMsuite (2025). at https://www.jcmwave.com/ URL.

[3] Shih, C.-W., Rodt, S. & Reitzenstein, S. Universal design method for bright quantum light sources based on circular Bragg grating cavities. *Optics Express* **31**, 35552–35564, doi: 10.1364/OE.501495 (2023).

[4] Rickert, L. *et al.* High Purcell enhancement in quantum-dot hybrid circular Bragg grating cavities for GHz clock rate generation of indistinguishable photons. *ACS Photonics* **12**, 464–475, doi: 10.1021/acsphotonics.4c01873 (2025).

[5] Peniakov, G. *et al.* Polarized and unpolarized emission from a single emitter in a bullseye resonator. *Laser & Photonics Reviews* **18**, 2300835, doi: 10.1002/lpor.202300835 (2024).

[6] Connors, E. J. *et al.* Low-frequency charge noise in Si/SiGe quantum dots. *Physical Review B* **100**, 165305, doi: 10.1103/PhysRevB.100.165305 (2019).

[7] Manna, S. *et al.* Surface passivation and oxide encapsulation to improve optical properties of a single GaAs quantum dot close to the surface. *Applied Surface Science* **532**, 147360, doi: 10.1016/j.apsusc.2020.147360 (2020).
